# MMKE: Multi-trial vector-based monkey king evolution algorithm and its applications for engineering optimization problems

**DOI:** 10.1371/journal.pone.0280006

**Published:** 2023-01-03

**Authors:** Mohammad H. Nadimi-Shahraki, Shokooh Taghian, Hoda Zamani, Seyedali Mirjalili, Mohamed Abd Elaziz

**Affiliations:** 1 Faculty of Computer Engineering, Najafabad Branch, Islamic Azad University, Najafabad, Iran; 2 Big Data Research Center, Najafabad Branch, Islamic Azad University, Najafabad, Iran; 3 Centre for Artificial Intelligence Research and Optimisation, Torrens University Australia, Adelaide, Australia; 4 Yonsei Frontier Lab, Yonsei University, Seoul, South Korea; 5 Department of Mathematics, Faculty of Science, Zagazig University, Zagazig, Egypt; Universidad de Guadalajara, MEXICO

## Abstract

Monkey king evolution (MKE) is a population-based differential evolutionary algorithm in which the single evolution strategy and the control parameter affect the convergence and the balance between exploration and exploitation. Since evolution strategies have a considerable impact on the performance of algorithms, collaborating multiple strategies can significantly enhance the abilities of algorithms. This is our motivation to propose a multi-trial vector-based monkey king evolution algorithm named MMKE. It introduces novel best-history trial vector producer (BTVP) and random trial vector producer (RTVP) that can effectively collaborate with canonical MKE (MKE-TVP) using a multi-trial vector approach to tackle various real-world optimization problems with diverse challenges. It is expected that the proposed MMKE can improve the global search capability, strike a balance between exploration and exploitation, and prevent the original MKE algorithm from converging prematurely during the optimization process. The performance of the MMKE was assessed using CEC 2018 test functions, and the results were compared with eight metaheuristic algorithms. As a result of the experiments, it is demonstrated that the MMKE algorithm is capable of producing competitive and superior results in terms of accuracy and convergence rate in comparison to comparative algorithms. Additionally, the Friedman test was used to examine the gained experimental results statistically, proving that MMKE is significantly superior to comparative algorithms. Furthermore, four real-world engineering design problems and the optimal power flow (OPF) problem for the IEEE 30-bus system are optimized to demonstrate MMKE’s real applicability. The results showed that MMKE can effectively handle the difficulties associated with engineering problems and is able to solve single and multi-objective OPF problems with better solutions than comparative algorithms.

## 1. Introduction

Metaheuristic algorithms are shown to be advantageous for addressing challenging optimization problems with diverse properties, including but not limited to high dimensionality, multimodality, and non-differentiability in a reasonable time [1]. This has led to the widespread use of these algorithms and an increasing trend of developing new and improved algorithms. Such techniques can be considered as approximate algorithms that are the successful alternative approach for solving problems in polynomial time. The stochastic nature of these algorithms keeps them distinct in comparison with deterministic methods or conventional optimization algorithms. Nature-inspired algorithms are a class of problem solvers inspired by nature’s natural phenomena to design new and robust competing algorithms. Taking into account the No-Free-Lunch (NFL) theorem [2], that asserts no single optimization algorithm can handle all problems of any complexity, numerous new optimization algorithms have been designed that are capable of handling the vast majority of optimization issues. Furthermore, the same algorithm is also observed to have variable outcomes when applied to the same problem depending on the values of the parameters.

Metaheuristic algorithms can be categorized as either population-based or single solution-based. The single solution-based algorithms are focused on exploiting and expanding a solution for crossing the local optimal point. In contrast to single-based metaheuristics, population-based metaheuristics begin with a collection of solutions known as population or swarm. They then construct a new population of solutions via an iterative process. This allows for the transmission of information between the solutions. In real-world issues, there is a strong likelihood that a single solution-based algorithm may become stuck on the local optima. On the other hand, population-based algorithms are known to be extremely exploratory of the search space, with less incidence of becoming stuck locally. If a solution traps within the local suboptimal, further iterations will help to avoid it by including alternative solutions. As a downside, these algorithms are computationally costly and need more objective function evaluations.

Nature-inspired metaheuristic algorithms typically draw their inspiration from natural phenomena such as biological, physical, chemical, and geological principles [3]. Nature-inspired algorithms can be classified into three categories based on the source of inspiration [4]: evolution-based, swarm intelligence-based, and physics-based algorithms. Evolution-based algorithms tend to mimic creatures’ evolutionary behavior concepts in nature. In this category some prominent algorithms are genetic programming (GP) [5], differential evolution (DE) [6], evolution strategy (ES) [7], genetic algorithm (GA) [8], and evolutionary programming (EP) [9]. Swarm-intelligence-based algorithms are inspired by the social and collective behavior of swarms in nature, such as colonies of bees and ants, animal herds, and birds’ flocks. Some of the prevailing and recently introduced algorithms in this category are particle swarm optimization (PSO) [10], ant colony optimization (ACO) [11], artificial bee colony (ABC) [12], symbiotic organism search (SOS) [13], salp swarm algorithm (SSA) [14], squirrel search algorithm (SSA) [15], crow search algorithm (CSA) [16], grey wolf optimizer (GWO) [17], capuchin search algorithm (CapSA) [18], and Snake Optimizer (SO) [19].

The third category is physics-based algorithms that are derived from the fundamental physical laws existing in nature. Some well-known algorithms in this category are simulated annealing (SA) [20], big bang-big crunch (BB-BC) algorithm [21], gravitational search algorithm (GSA) [22], water evaporation optimization (WEO) [23], and Archimedes optimization algorithm (AOA) [24].

The monkey king evolution (MKE) [25] is one of the algorithms in the evolution-based category inspired by a Chinese mythological novel. The population is guided in this population-based algorithm by the best monkey king of the whole population. The MKE algorithm is prone to premature convergence and insufficient balance between exploitation and exploration. These shortcomings originate from the MKE’s evolution scheme (search strategy) that updates all monkey kings’ positions by considering the best one and using a fixed control parameter. Moreover, the MKE algorithm has a single evolution strategy (search strategy) to deal with various kinds of problems that results in inefficient performance when confronted with various issues. Thus, by incorporating multi-evolution strategies into the MKE algorithm, we aim to make it more effective in solving a wide variety of real-world optimization problems, which is the primary goal of this work.

As part of our prior work, we introduced a multi-trial vector (MTV) approach [26] to leverage a mix of evolution strategies and handle various issues. This approach comprised four components: winner-based distributing, multi-trial vector producing, evaluating and population updating, and life-time archiving. The MTV approach has the merits of introducing and combining multiple search strategies incorporated by defining distribution policies over the population, which increases the algorithms’ performance. Using the MTV approach can fulfill the need to define different strategies that can be adapted to various characteristics of problems in every stage of the search process to avoid local optima entrapment, prevent premature convergence, and strike an appropriate balance between exploitation and exploration. It is our motivation to use the merits of the MTV approach and a combination of three trial vectors to significantly improve the MKE algorithm’s performance and handle various complex real-world optimization issues.

In this paper, we propose an effective multi-trial vector-based monkey king evolution (MMKE) algorithm using the multi-trial vector (MTV) approach [26]. In the design of the multi-trial vector producing step of the MTV approach, novel best-history trial vector producer (BTVP) and random trial vector producer (RTVP) are introduced to cooperate with canonical MKE (MKE-TVP). Each trial vector producer (TVP) is adjusted to maintain a particular search behavior during the process of solving different problems with diverse characteristics. Also, each TVP is used to apply to a section of the population that is dedicated to that TVP. Through the winner-based MTV distribution strategy, the percentage of the devoted population is adjusted at consistent intervals depending on the number of individuals each TVP improved. The integration of different evolution strategies in the MTV approach can improve the balance between exploration and exploitation, prevent premature convergence, and avoid local and deceptive optimum conditions. As part of the validation of the proposed MMKE algorithm, experiments on 29 test functions taken from the CEC 2018’s special session on real-parameter optimization [27] were conducted. An evaluation of the results was made in comparison with state-of-the-art evolutionary and swarm intelligence algorithms, and then the statistical analysis was done. Additionally, the applicability of MMKE was demonstrated by solving engineering problems. Based on the comparisons and statistical analyses, the MMKE algorithm has proven to be superior to comparative algorithms.

The general methodology used in this research includes problem modeling and mathematical formulation, algorithmic design and development, performance assessment and comparison, and deployment for real-world applications. Regarding problem modeling and mathematical formulation, optimization is to find the best solution(s) from a collection of solutions that minimize (or maximize) an objective function and adhere to a number of constraints. The task of optimization can be mathematically formulated as a search to find X^*^, which minimizes such an objective function f(X^*^) < f(*X*) for all *X* ∈ *Ω*, where *Ω* is a non-empty large finite set as the domain of the search. Next, in the algorithmic design and development, the proposed MMKE algorithm’s methodology is initializing, winner-based distributing, control stopping criteria, multi-trial vector producing, evaluating and population updating, and archiving.

The performance assessment in this research is done the same as works on metaheuristics which is qualitative and quantitative. In the qualitative assessment, a visual analysis of MMKE is provided along with two directions: trial vector impact analysis and exploration and exploitation behavior analysis. Then, MMKE’s performance was evaluated using a quantitative analysis including exploration and exploitation, local optima avoidance, and convergence evaluation compared to eight state-of-the-art metaheuristic algorithms. Eventually, deployment for real-world applications includes five engineering problems that were used to study further the MMKE algorithm’s potential to address real-world engineering difficulties.

The contributions of this study can be summarized as follows:

- Amending the MKE algorithm’s evolution scheme with the multi-trial vector (MTV) approach to enhance the original MKE’s performance.
- Dividing the MKE’s population into the number of sub-populations based on the winner-based distribution policy and using an exclusive TVP for each sub-population to guide the individuals.
- Proposing northward evolution strategies by introducing two new trial vector producers.
- Using a combination of best-history trial vector producer (BTVP) and random trial vector producer (RTVP) in conjunction with canonical MKE (MKE-TVP) to improve global search capability, reducing the risk of trapping in the local optima and preventing premature convergence of the original MKE.

Based on the mentioned deficiencies of the MKE algorithm and the merits of the MTV approach, the research hypothesis is stated as follows: the performance of the MKE algorithm in terms of accuracy of the gained results, avoidance of local optima trapping, equilibrant exploitation and exploration, and prevention of premature convergence can be increased and enhanced using the MTV approach and effective trial vector producers.

The remainder of this article is structured in such a way that Section 2 summarizes relevant works. Section 3 contains the MKE algorithm’s mathematical model and flowchart. The proposed MMKE algorithm is detailed in Section 4. Section 5 discusses the MMKE’s qualitative and quantitative analysis, whereas Section 6 discusses the MMKE’s statistical analysis. MMKE’s applicability for solving real engineering design problems is assessed in Section 7. Finally, Section 8 concludes research findings and recommends further studies.

## 2. Related work

Nature-inspired algorithms have become popular choices to solve a wide variety of optimization issues in diverse areas such as engineering [28–34], image processing and segmentation [35–37], global optimization [38–45], software fault prediction [46], scheduling [47–50], photovoltaic modeling [51–54], structural design problems [55–59], power and energy management [60–62], planning and routing problems [63–65], power take off and placements of wave energy converters [66, 67], power consumption [68, 69], and wind speed prediction [70, 71]. Although the majority of nature-inspired algorithms are proposed to solve continuous problems, there have been various methods to adapt these algorithms to solve problems with discrete nature [72]. Numerous real-world issues have been solved using the adapted methods, including feature selection [73–79], clustering and community detection [80–84], and medical diagnosis [85–87].

In nature-inspired algorithms, a population of individuals searches different regions of the solution space cooperatively by applying a search mechanism derived from natural phenomena. These algorithms possess two substantial aspects exploitation and exploration. Exploration is related to an optimization algorithm’s capability to explore diverse areas of the search space on a global scale. In contrast, exploitation refers to an algorithm’s capability to identify solutions that are close to the optimal solution in promising regions. An excessive level of exploration can lead to a decrease in the probability of finding the optimal solution. At the same time, too much exploitation can cause the algorithm to be trapped in local optima [88]. Thus, a proper balance between exploration and exploitation is essential to enhance the search ability, avoid falling into local optima, and achieve a reasonable solution.

Although these aspects are considered in the design of metaheuristic algorithms, different behaviors can occur when they confront various optimization problems. Thus, different alterations are also performed in the canonical versions of nature-inspired algorithms to solve optimization problems with different characteristics and complexities. As a clarification, imbalanced exploration and exploitation of the GWO algorithm is improved in the Gaze cues learning-based grey wolf optimizer (GGWO) [89]. The same issue is resolved by defining a hybrid phase into the MFO algorithm in the variant improved moth-flame optimization (IMFO) algorithm [90]. Likewise, the search ability of the basic EPO algorithm is improved for color image segmentation in improved emperor penguin optimization (IEPO) [91] by using the levy flight, Gaussian mutation, and opposition-based learning. Higher performance and quick convergence are achieved by a new parameter-adaptive DE (PaDE) algorithm [92] while solving numerical optimization problems.

**Fig 1:**
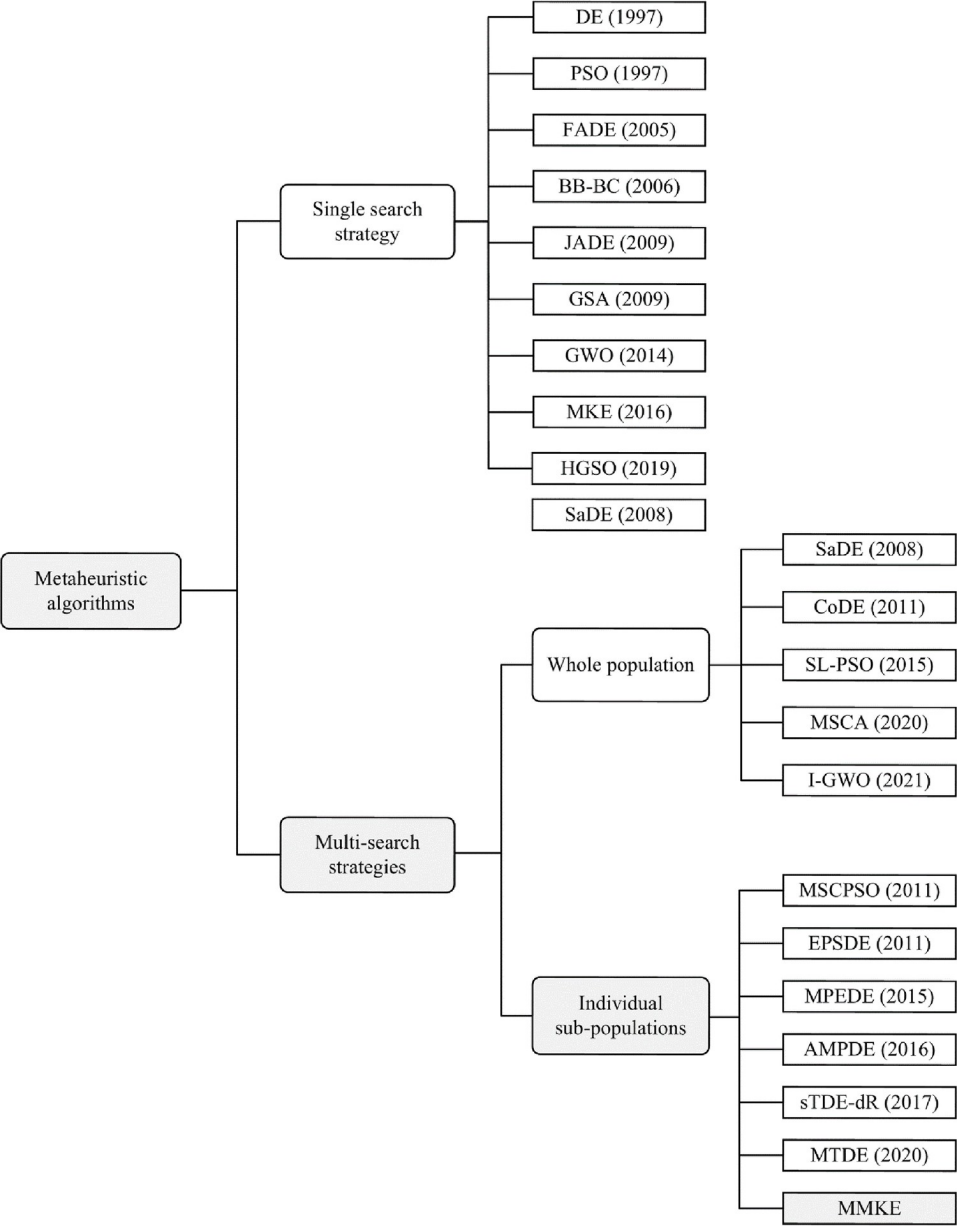
The classification of single and multi-search strategies.

In stochastic search algorithms, the quality of obtained solutions depends on various aspects, such as the search strategy, the adjustment of the parameters, and constraint handling of the problem. As shown in Fig 1, metaheuristic algorithms based on the number of strategies used during the search process can be classified into single search strategy algorithms and multi-search strategies algorithms. Single search strategy algorithms may not be able to find an appropriate solution for problems with complex search space because an adaptation needs with the changes in the search landscape during the optimization process. Multiple strategies have different characteristics and capabilities in multi search strategies algorithms, such as exploration, exploitation, and maintaining diversity. Thus, an effective algorithm with a combination of the different strategies has the potential to deal with diverse kinds of optimization problems. Moreover, it is beneficial to use different search techniques to increase the probability of locating the optimal solution for a sophisticated optimization issue with a complex search space.

As shown in Fig 1, the algorithms containing multi-search strategies can be applied to the whole population or individual sub-populations. In the first category, all the strategies are applied to the whole population to discover the survivors for the following iteration. To be more specific, algorithms such as SaDE [93], CoDE [94], SL-PSO [95], I-GWO [96], and MSCA [97] are kinds of algorithms that apply multi-search strategies to the whole population of individuals. It is an effective way to deal with different problems but computationally expensive as needs to evaluate the fitness value of multiple produced candidates. Qin et al. have developed a self-adaptive DE (SaDE) that effectively uses two mutation strategies concurrently by adjusting the control parameters F and CR in accordance with prior knowledge. Wang et al. suggested a composite DE (CoDE) that includes three trial vector techniques and three control parameter settings. In order to create new vectors, each individual is assigned a new search strategy and parameter selection at random. Cheng and Jin proposed a social learning PSO (SL-PSO) in which all the particles except the best one use a social learning mechanism to learn from particles with better objective value in the current fitness-sorted population. In [97], a multi-strategy enhanced sine cosine algorithm named MSCA is proposed, in which four search mechanism is applied to the search agents, and in each iteration, the previous position replaces the best candidate solutions. In [96], I-GWO was proposed by introducing a new search strategy named dimension learning-based hunting (DLH). The I-GWO enhances diversity, strikes a balance between exploration and exploitation, and copes with premature convergence of GWO. The wolves’ new position is selected between the GWO or the DLH search strategies according to the quality of obtained solutions.

In the second category, the main population is always split up into some small sub-populations, each of which is updated according to the assigned search strategy and control parameters to generate candidate solutions. This is a potential way to improve optimization performance by when each sub-population is responsible for exploring, exploiting, maintaining diversity, and reducing the probability of trap in local optima. In the literature, some algorithms were developed with multi sub-populations, each of which uses a different search strategy. In [98], a multi-swarm cooperative particle swarm optimizer named MCPSO was proposed in which the population contains a master swarm and several slave swarms. The particles of the master swarm move according to both master swarm and slave swarms’ knowledge, while the particles of slave swarms move based on the independent execution of PSO variants. In this algorithm, slave swarms can preserve diversity, and the interaction of this two kind of swarms has an effect on the balance between exploration and exploitation. In [99], multi-strategy ensemble particle swarm optimization (MEPSO) was proposed, in which parts Ⅰ and Ⅱ are a division of particles into two parts. Each part had a distinctive role in the search process; indeed, the Gaussian local search and differential mutation strategies were utilized in combination with the traditional PSO search algorithm for each part. The investigation revealed that the method utilized in part I can improve the algorithm’s convergence, while the other part can improve the algorithm’s exploration capability and avoidance of local optima.

EPSDE was suggested in [100], which comprises a collection of mutation and crossover and a pool of values matching to each related parameter that strive to develop better candidate solutions. EPSDE has proven to be competitive on a variety of optimization problems due to its proper moves in the search space. In [101], MPEDE, a multi-population based DE was proposed in which the population is partitioned into three sub-populations, each using a different mutation and update strategy. The constituent mutation strategies were selected from the literature are competitive in solving unimodal and multimodal optimization problems and performing a better exploration. The experimental results on the benchmark functions demonstrated that the proposed algorithm outperformed the other DE variants. An adaptive multi-population differential evolution (AMPDE) algorithm was proposed in [102], in which the size of the sub-populations was adaptively altered considering information gathered from prior search knowledge. Individuals from each sub-population were modified in accordance with the crossover operator that was assigned to them from GAs in order to create perturbed vectors. In [103], an adaptive DE with dynamic population reduction was proposed named sTDE-dR. The entire population was clustered into multiple tribes with different sizes, and each tribe has different mutation and crossover strategies. The experimental results showed the robustness of the proposed algorithms in comparison to the other comparative algorithms. As a variation on the developed MTV approach, [26], proposed a multi-trial vector-based differential evolution algorithm named MTDE. Despite previous algorithms that populations were divided into multiple sub-populations with smaller sizes, in the MTDE algorithm, the whole population is divided into three sub-populations with different sizes based on the defined distribution policy. The aim of using the combination of TVPs in the proposed algorithm is to maintain the population diversity, the balance between the exploration, and enhance the local search ability.

## 3. Monkey King Evolution (MKE) algorithm

The monkey king evolution algorithm (MKE) is a simple evolutionary algorithm inspired by the monkey king, a character of a Chinese mythological novel named “Journey to the West”. In a tough situation, the monkey king can transform into a number of small monkeys, each of which tries to find and report a solution. Then, the monkey king selects the most suitable solution for the trouble. In the MKE algorithm, *N* monkey kings are randomly distributed in the search space with *Dim* dimensions. The monkey kings’ position and the i^th^ monkey king are denoted by matrix *X* and its i^th^ row *X*_*i*_ *= {x*_*i*,*1*_, *x*_*i*,*2*_, *…*, *x*_*i*,*Dim*_*}*. Then, in each generation, the monkey kings’ fitness is calculated to determine the gbest particle with the best fitness value and then the matrix *X*_*gbest*_ is built in which all rows are replicated by gbest position.

The MKE uses an evolution scheme to update the monkey kings’ position in which first, two different matrixes *X*_*r1*_ and *X*_*r2*_ are constructed by permuting the row vectors of the matrix *X*. Next, the matrix of mutated monkeys denoted by *B* is built using Eq (1) where *FC* is the fluctuation coefficient parameter with a constant value 0.7.


$$B=X_{gbest}+FC\times (X_{r1}-X_{r2})$$ (1)

Then, the evolved monkeys are calculated by Eq (2), where *M* is a transformation matrix, and $\bar{M}$ is the binary inverse of *M*. The matrix *M* is generated from a lower triangular matrix with the elements set by one. After that, each row of the matrix’s elements is randomly permutated, and then the sequence of row vectors of the matrix is separately permuted.


$$X=M\times X+\bar{M}\times B$$ (2)

**Fig 2:**
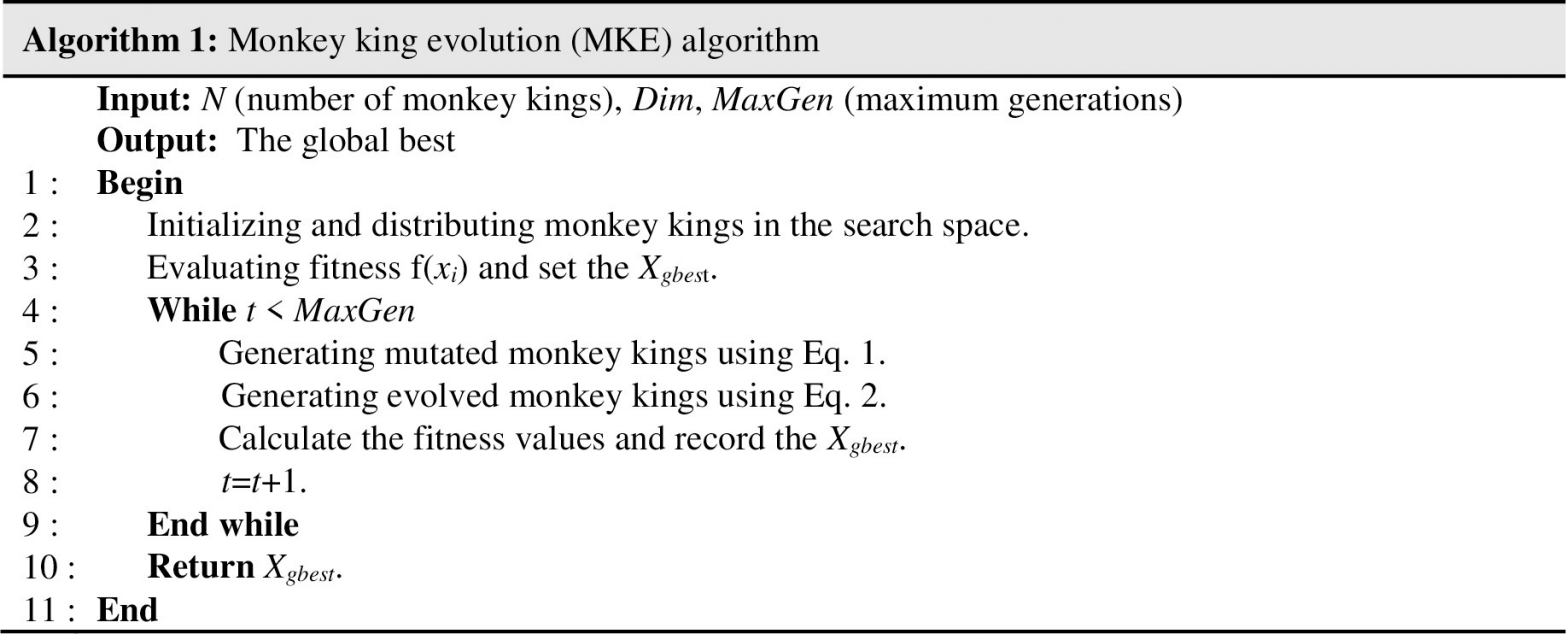
Pseudo-code of MKE.

Finally, as shown in Fig 2, in the 7^th^ line of the pseudo-code of MKE, each evolved monkey’s fitness value is calculated by which either the particle at the current position or its trial vector is selected to survive to the next generation.

Although the benchmarked results show that the MKE algorithm’s performance is sufficient in comparison to some PSO-based variants, it has shortcomings, including premature convergence and inadequate exploration/exploitation balance. These defects originate from updating all monkey kings’ positions using the MKE’s evolution scheme based on the gbest.

## 4. Multi-trial vector-based monkey king evolution (MMKE) algorithm

Solving different optimization problems with various characteristics such as uni/multi-modality, (non)separability, (a)symmetry [27, 104] requires suitable search strategies. Furthermore, maintaining an equilibrium between exploration and exploitation prevents premature convergence and stagnation and provides a higher level of population diversity. Then, the effectiveness of a metaheuristic algorithm in solving optimization problems depends on selecting an appropriate search strategy and setting its parameters. On the other hand, based on the no-free-lunch (NFL) theorem, there is no general-purpose search strategy to cope with optimization problems and different strategies are required for solving diverse problems [2]. These considerations led us to propose an improved variant of the MKE algorithm named multi-trial vector-based monkey king evolution (MMKE) algorithm to tackle its insufficiencies. MKE’s shortcomings include a premature convergence to local optima, an improper balance between exploration and exploitation, and a fixed control parameter. These issues suppress the MKE algorithm’s ability to handle different complicated problems.

**Fig 3:**
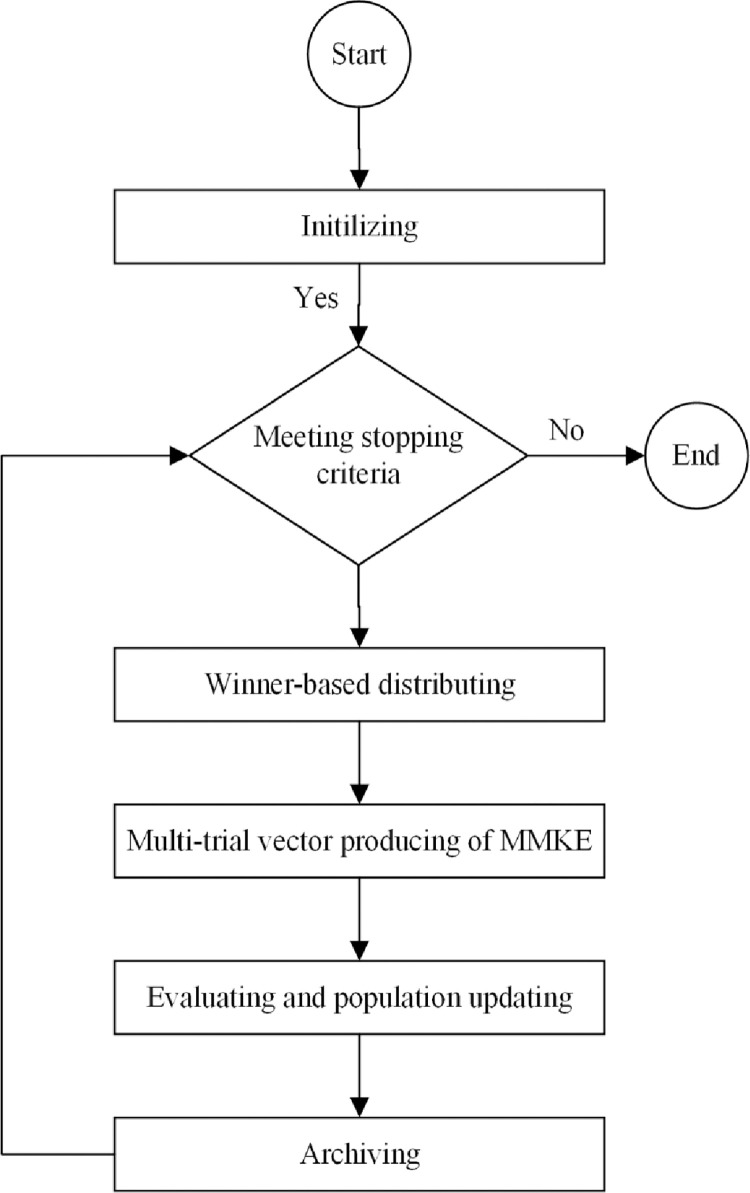
The framework of the proposed MMKE algorithm.

As shown in Fig 3, the proposed MMKE algorithm’s framework consists of four main phases: initializing, winner-based distributing, multi-trial vector producing of MMKE, evaluating and population updating, and archiving. Each of these phases are explained in the following paragraphs.

Table 1 provides a nomenclature to show the parameters’ description used in the following sections.

**Table 1:** The nomenclature used in the MMKE algorithm.

Parameter	Description
*X*	The monkey king’s position matrix
*X*_*gbest*_	The replicated matrix of the gbest
*FC*	The fluctuation coefficient parameter
*M*, $\bar{M}$	The transformation matrix and its reverse
*N*_*MKE-TVP*_, *N*_*BTVP*_, and *N*_*RTVP*_	The portion size of MKE-TVP, BTVP, and RTVP
*X*^*Mpop*^, *X*^*Bpop*^, and *X*^*Rpop*^	The sub-population of each TVP
*U*^*Mpop*^, *U*^*Bpop*^, and *U*^*Rpop*^	The candidate sub-population of each TVP
*gbest* ^*pop*^	The best monkey from *X*
*X* ^*BHpop*^	Replicated matrix of the best-history archive
*X* ^*Allpop*^	The union population of *X* and archive

The simple evolution scheme of the MKE algorithm is substituted by the MTV approach to boost its performance using different evolution strategies for solving various optimization problems. Adapting the MTV approach enables the MKE algorithm to use a varying number of trial vector producers (TVPs) as required to achieve a particular behavioral outcome. Moreover, another advantage of using the MTV approach is to dedicate a portion of the whole population based on the winner-based distribution policy to each TVP. Furthermore, the population exchange between TVPs can enhance information sharing between monkeys and maintain diversity. The proposed MMKE algorithm uses three different evolution strategies integrated with a random fluctuation coefficient for enhancing the global search ability, reducing the probability of trapping in local optima, and preventing the original MKE’s premature convergence.

**Fig 4:**
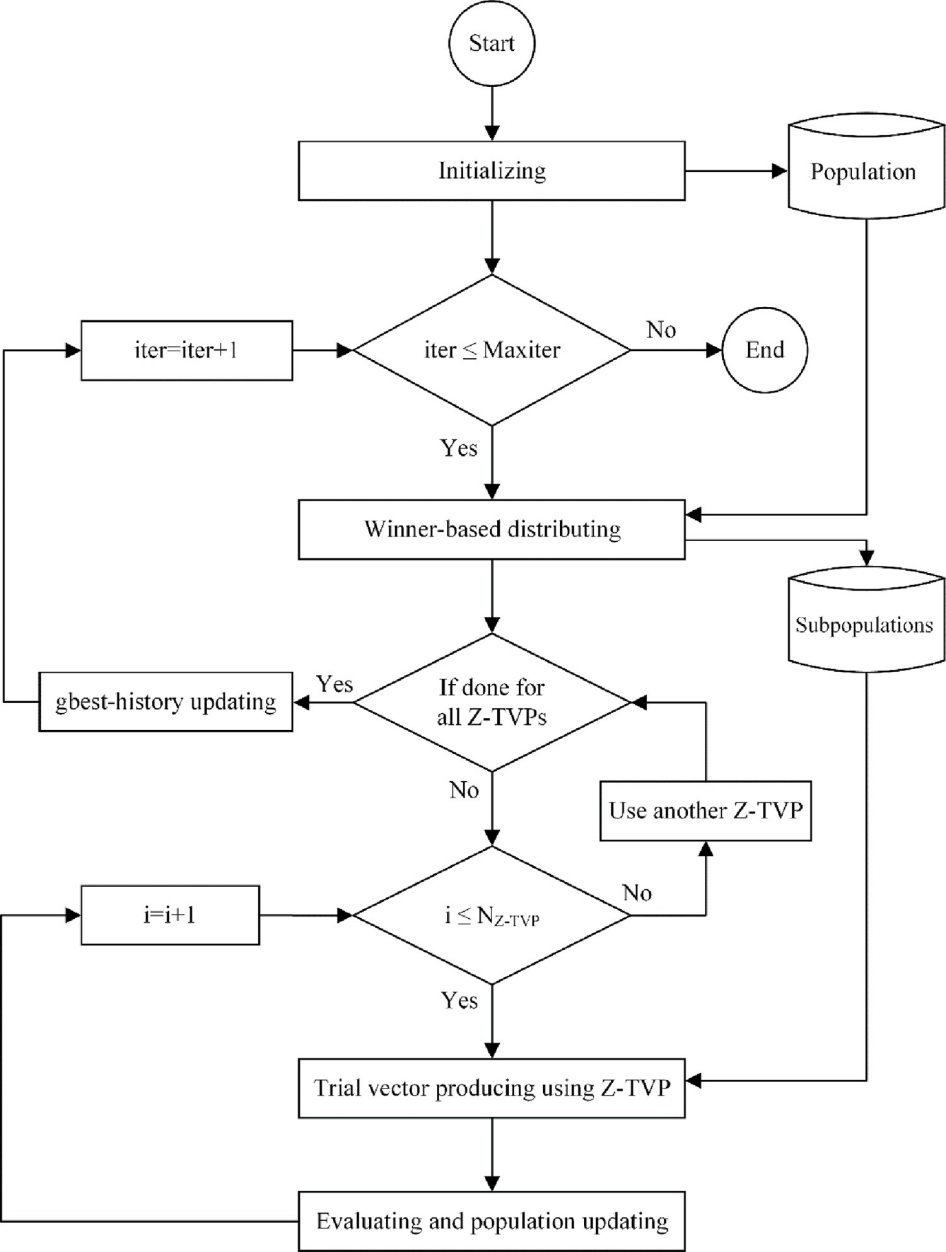
The flowchart of the proposed MMKE algorithm.

The proposed MMKE algorithm’s flowchart is depicted in Fig 4, consisting of initializing, winner-based distributing, multi-trial vector producing, evaluating and population updating, and archiving. In the initializing step, after the random distribution of *N* monkeys and evaluating the fitness of the initial population in every *ngen* generation and in the winner-based distributing step, the sub-population size of TVPs is determined by considering the reward rule distribution policy. Then, three trial vector producers, canonical MKE (MKE-TVP), best-history trial vector producer (BTVP), and random trial vector producer (RTVP), cooperate in the step of multi-trial vector producing to guide the monkeys over the search space. MMKE’s ability to detect promising regions when solving different problems is significantly facilitated when these TVPs are combined. Then, in evaluating and population updating step, the monkeys’ current position is updated after calculating the fitness of evolved monkeys. According to the archiving step, the inferior ones are archived to use their knowledge in TVPs. The step-wise procedure of the proposed MMKE algorithm is explained as follows.

**Initializing step:** *N* monkeys are randomly overspread within the predefined range [*l*, *u*] using Eq (3).


$$x_{ij}=l_{j}+(u_{j}-l_{j})\times rand(0.1)$$ (3)

Where *x*_*ij*_ is the position of the i^th^ monkey king in the j^th^ dimension, *l*_*j*_ and u_j_ are j^th^ dimension’s lowest and maximum bounds, and rand represents a random value between 0 and 1, respectively. The positions of *N* monkeys are stored in matrix *X*, which is a *N×Dim* matrix. The fitness value of monkey *X*_*i*_ in t^th^ generation is calculated by f (*X*_*i*_ *(t)*).

**The winner-based distributing step:** The whole generation is divided into *k* portions including *ngen* generations. The first step of each portion is to select the best TVP or the producer with the highest rate of improvement over the previous *ngen* generations. Therefore, the improved rate of each TVP, IR_Z-TVP_ (Z represents one of the TVPs), is calculated by dividing the number of improved monkeys by the number of function evaluations in the previous portion using Eq (4).


$${IR}_{Z-TVP}=\frac{\#improved\;solutions}{\#function\;evaluations}$$ (4)

After determining the improved rate of each TVP, the size of each TVP’s sub-population is calculated for the next *n* generations using the reward rule distribution policy defined in Eq (5),

$$N_{Z-TVP}=\left\{ \begin{array}{ll} 2\times \lambda \times N & for\;TVP\;with\;higher\;improved\;rate\\ \lambda \times N & for\;other\;TVPs \end{array}\right.$$ (5)

where there is *N* number of monkeys, *N*_*Z-TVP*_ is the size of the sub-population considering the TVPs’ improved rate, and λ = 0.25.

**Multi-trial vector producing step:** In each generation, monkey *X*_*i*_ is moved by one of the three different TVPs, MKE-TVP, BTVP, or RTVP. The MKE-TVP facilitates exploitation capability by enabling individuals to search for new solutions in their locality or immediate vicinity. Exploitation and escape from the local optima are handled using BTVP, whereas RTVP is designed to balance exploration and exploitation. Once each TVP mutates its dedicated sub-population, the evolved vector of the monkeys is produced using the *M* and $\bar{M}$ matrixes by Eq (6).

$$U_{i}^{Mpop}(t+1)=M_{i}\times X_{i}^{Mpop}+{\bar{M}}_{i}\times V_{i}^{Mpop}$$

$$U_{i}^{Bpop}(t+1)=M_{i}\times X_{i}^{Bpop}+{\bar{M}}_{i}\times V_{i}^{Bpop}$$ (6)

$$U_{i}^{Rpop}(t+1)=M_{i}\times X_{i}^{Rpop}+{\bar{M}}_{i}\times V_{i}^{Rpop}$$

Where *U*_*i*_^*Mpop*^, *U*_*i*_^*Bpop*^, and *U*_*i*_^*Rpop*^ indicate the produced candidate solution for *X*_*i*_^*Mpop*^, *X*_*i*_^*Bpop*^, and *X*_*i*_^*Rpop*^, i^th^ monkey of sub-population MKE-TVP, BTVP, or RTVP. *V*_*i*_^*Mpop*^, *V*_*i*_^*Bpop*^, and *V*_*i*_^*Rpop*^ indicate the mutated vector which generated for the i^th^ monkey of sub-population MKE-TVP, BTVP, or RTVP.

*Monkey king evolution trial vector producer (MKE-TVP)*: As mentioned in the preceding section, in each generation, the best monkey from *X* is considered as gbest and preserved in *gbest*^*pop*^. Next, to move the monkey *X*_*i*_ from *X*^*Mpop*^ sub-population, the constant fluctuation coefficient (*FC* = 0.7) multiples to the differentiate of the two randomly selected monkeys *X*_*r1*_^*Mpop*^ and *X*_*r2*_^*Mpop*^. Finally, the MKE-TVP generates the mutated vector *V*_*i*_^*Mpop*^ by Eq (7).


$$V_{i}^{Mpop}(t+1)={gbest}^{pop}(t)+FC\times \left(X_{r1}^{Mpop}(t)-X_{r2}^{Mpop}(t)\right)$$ (7)

Where *V*_*i*_^*Mpop*^ is the mutated vector for i^th^ monkey of *X*^*Mpop*^, *gbest*^*pop*^ indicates the best monkey from *X*, and *X*_*r1*_^*Mpop*^ and *X*_*r2*_^*Mpop*^ are two randomly selected monkeys from *X*^*Mpop*^ sub-population.

**Fig 5:**
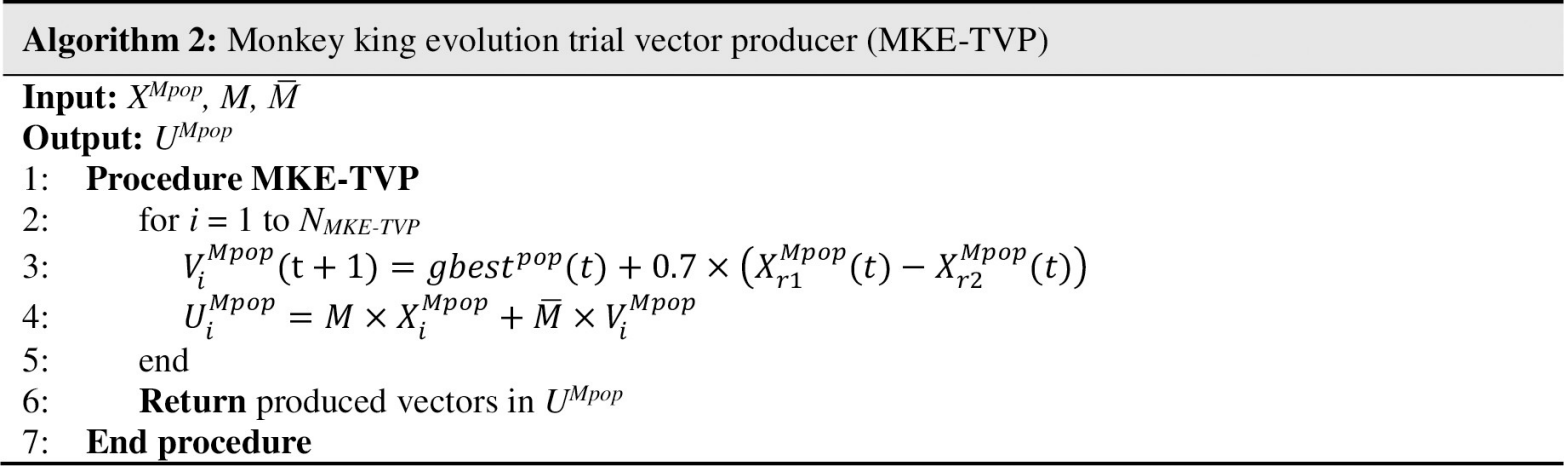
Pseudo-code of MKE-TVP.

As shown in Fig 5, this strategy is more explorative at the earlier stages of the evolution and afterward becomes more exploitative at the later stages of the optimization and will produce well-distributed solutions around the best monkey. Since the strategy used is the same as the MKE evolution scheme, this leads to produce identical monkeys throughout the generations, resulting in the occurrence of undesirable convergence.

**Fig 6:**
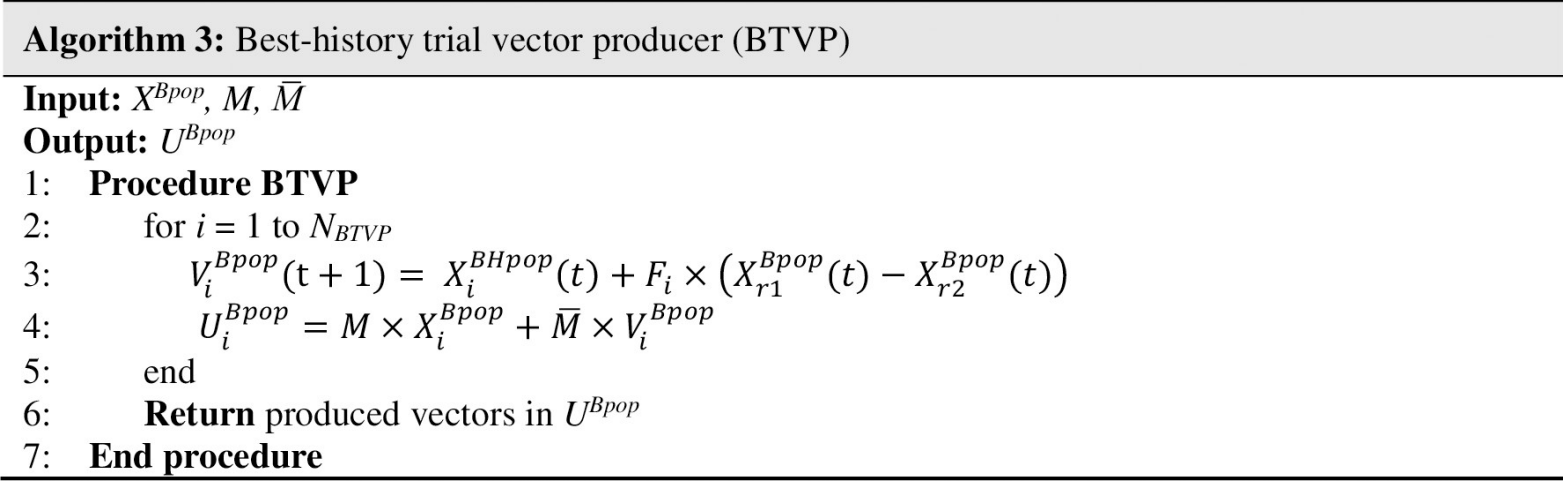
Pseudo-code of BTVP.

*Best-history trial vector producer (BTVP)*: Despite the widespread use of the best monkey in MKE-TVP to help for fast convergence, it might suffer from the premature convergence problem. Therefore, in BTVP, we aim to use the top best monkeys to direct the population’s evolution rather than the one global best. For this purpose, the best-history archive is designed to keep *M* recent best monkeys. At first, the best-history archive is initialized with the gbest vector and then the best vector is distinctively added to the gbest-history in the subsequent generations. If the best-history archive has no entry, the current best is replaced by the entry with the worst fitness value. In each generation, a matrix *X*^*BHpop*^ which has *N*_*BTVP*_ rows and *Dim* columns is created by repeating the best-history for (*N*_*BTVP*_*/M*) times. Then, the mutated vector *V*_*i*_^*Bpop*^ is produced by Eq (8),

$$V_{i}^{Bpop}(t+1)=X_{i}^{BHpop}(t)+C\times \left(X_{r1}^{Bpop}(t)-X_{r2}^{Bpop}(t)\right)$$ (8)

where *V*_*i*_^*Bpop*^ is the mutated vector for i^th^ monkey of *X*^*Bpop*^, *X*_*i*_^*BHpop*^ is the i^th^ row of the best-history population and *X*_*r1*_^*Bpop*^ and *X*_*r2*_^*Bpop*^ are two randomly selected individuals from *X*^*Bpop*^. Parameter *C* is a decreasing coefficient [105] which is computed by Eq (9),

C=α−(α−β)×(((MaxGen−gen)/MaxGen))^μ (9)

where *α* and *β* are the initial and final values of parameter *C*, *MaxGen* and *gen* indicate the maximum number of generations and the current generation, and *μ* is a dimension dependent value. This strategy is less greedy than the MKE-TVP and prevents local optima trapping. The pseudo-code of the BTVP shown in Fig 6.

*Random trial vector producer (RTVP)*: RTVP is proposed to prevent premature convergence and keep exploration and exploitation in balance. In fact, these problems are caused by the inability of the evolution strategies to generate newly evolved vectors and the failure to produce new promising monkeys. Therefore, the last TVP uses the difference between the current monkey and a random one from the population and a difference between two random monkeys from its sub-population (*X*^*Rpop*^) and the combination of the archive and whole population (*X*^*Allpop*^). Then, the mutated vector *V*_*i*_^*Rpop*^ is produced by Eq (10).


$$V_{i}^{Rpop}(t+1)=X_{i}^{Rpop}(t)+F_{i}\times (X_{r1}^{Rpop}(t)-X_{i}^{Rpop}(t))+F_{i}\times (X_{r2}^{Rpop}(t)-X_{j}^{Allpop}(t))$$ (10)

Where *V*_*i*_^*Rpop*^ is the mutated vector for i^th^ monkey of *X*^*Rpop*^, *X*_*r1*_^*Rpop*^ and *X*_*r2*_^*Rpop*^ are two randomly selected individuals from *X*^*Rpop*^, *X*_*i*_^*Rpop*^ is the i^th^ monkey of *X*^*Rpop*^, and *X*_*j*_^*Allpop*^ is the randomly selected solution from the union of *X* and archive, respectively. As explained in the following, *F*_*i*_ is a scale factor which generated by Cauchy distribution.

Despite using the constant *FC*, for each monkey, a random number generated by Cauchy distribution [106] is calculated as *F*_*i*_ *= randc*_*i*_ *(μf*, *σ)*, where *μ*_*f*_ is the mean value of improved scale factors initiated by 0.5 and *σ* = 0.2. In order to determine the value of *F*_*i*_, it must lie within the range (0, 1]; if *F*_*i*_ is greater than 1, it is considered to be 1; otherwise, it should be recalculated. The scale factor *F* remains unchanged if there is absolutely no monkey with improved fitness in the population; however, if a monkey with enhanced fitness exists, μ_f_ is calculated using the weighted Lehmer mean by Eq (11).


$$\mu_{f}=\frac{\sum_{f_{i}\in S_{f}}{w_{f_{i}}*f}_{i}^{2}}{\sum_{f_{i}\in S_{f}}w_{f_{i}}*f_{i}}$$ (11)

Where *S*_*f*_ is the set of all scale factors of *X*_*i*_ that f(*X*_*i*_*(t+1)*) < f(*X*_*i*_*(t)*), and the weight *w*_*fi*_ is calculated by Eq (12), where $\Delta f_{i}=f(X_{i}(t))-f(X_{i}(t+1))$.


$$w_{f_{i}}=\frac{\Delta f_{i}}{\sum_{f_{i}\in S_{f}}\Delta f_{i}}$$ (12)

**Fig 7:**
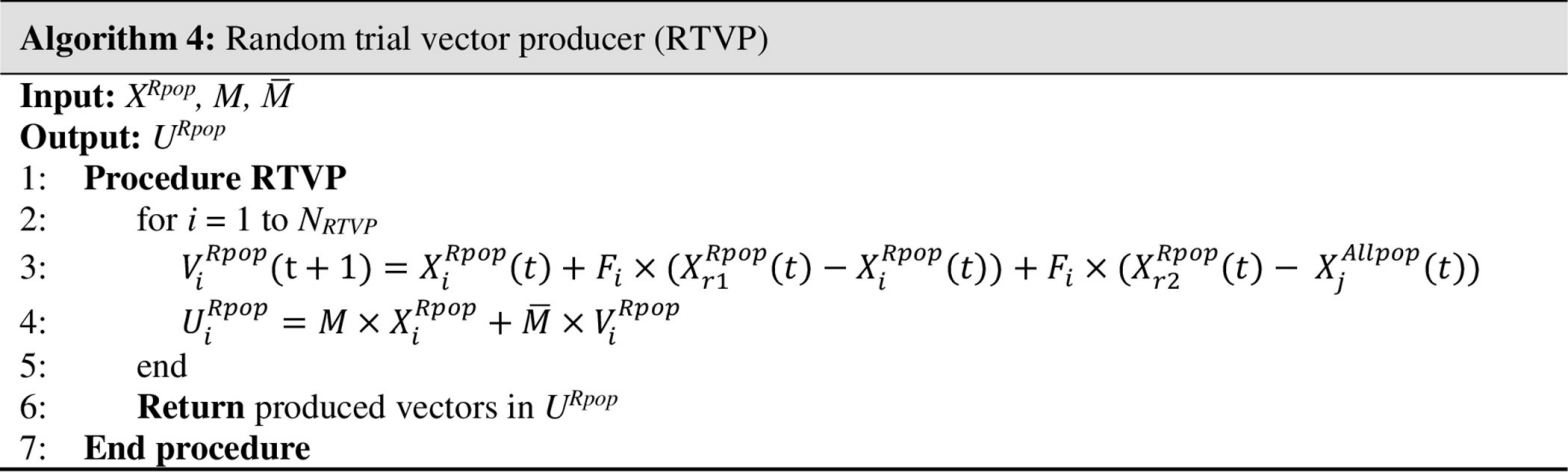
Pseudo-code of RTVP.

This TVP has a top priority for exploring and leading the search to the global optimum. Differences between random solutions are utilized to balance exploration and exploitation and preserve diversity throughout the optimization. The RTVP’s pseudo-code shown in Fig 7.

**Evaluating and population updating:** After the evolution of one generation of monkeys, the evolved monkeys’ fitness value is assessed and compared with that of the previous generation and the best monkeys are survived and are allowed to participate in the next generation.

**Archiving:** During each generation, inferior monkeys, the solutions that their candidates were replaced, possess important information about the search space’s potential areas. Thus, it is useful to save and distribute their knowledge in order to advise future generations of monkeys. To prohibit the residency of the earlier inferior monkeys’ in the archive, each of them has a lifetime variable that demonstrates the duration of being in the archive. At the end of each generation, the inferior monkeys are added to the archive, and the lifetime is increased by one. The number of monkeys in the archive must not exceed *N*; in that case, inferior monkeys with a longer lifespan should be removed in random order. Using the archive throughout the development of monkeys makes it possible to retain a high level of diversity in simple and complex problems. The proposed MMKE algorithm’s pseudo-code is demonstrated in Fig 7.

### 4.1 The computational complexity of MMKE

**Fig 8:**
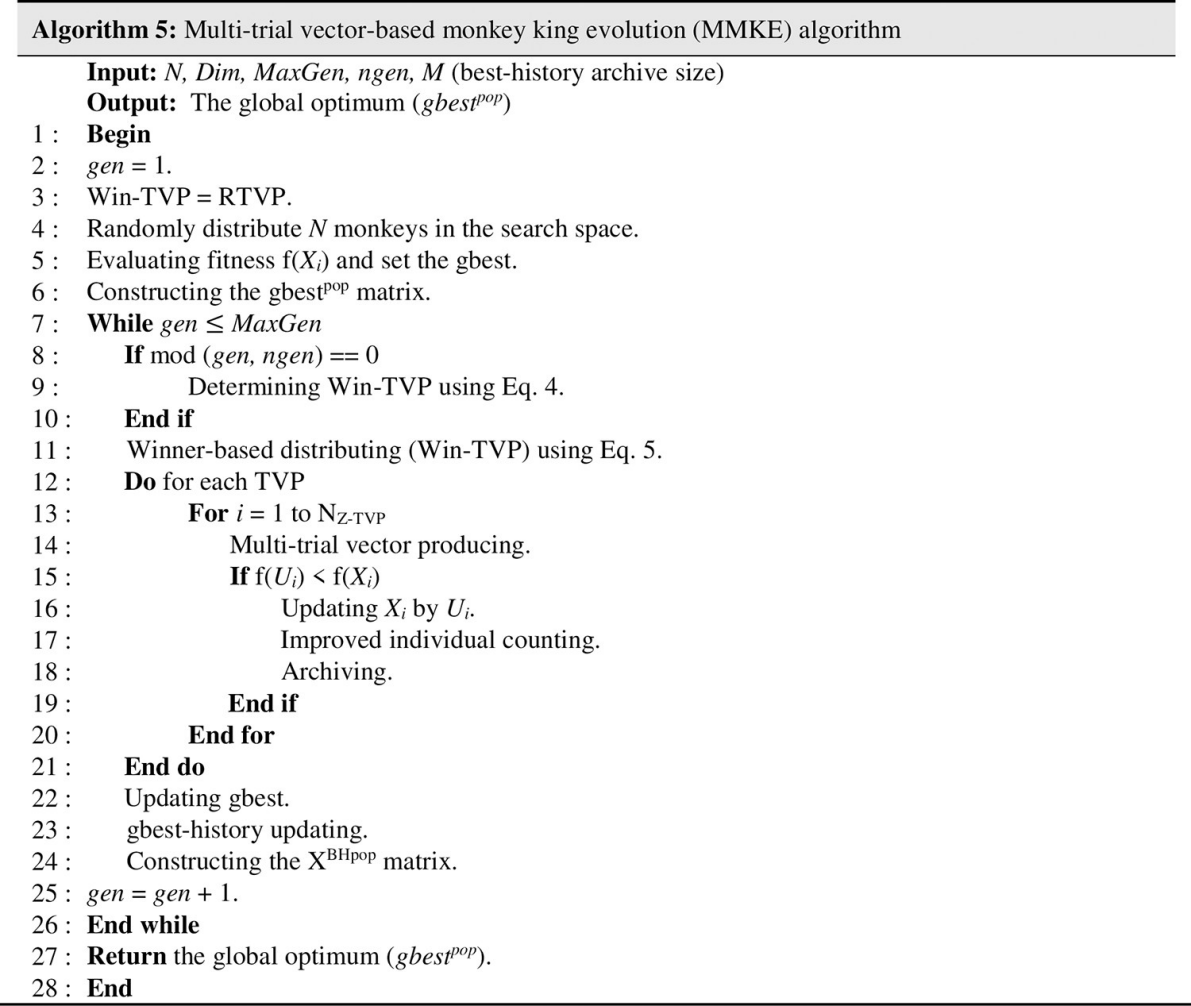
Pseudo-code of MMKE.

As shown in Fig 8, the main steps of the MMKE algorithm are initializing, winner-based distributing, multi-trial vector producing, and evaluating and population updating. All *N* monkeys are distributed in the D-dimensional search space in the first step with computational complexity O(ND). Then, the complexity of the while-loop (lines 7–26), including the winner-based distributing step (line 11), multi-trial vector producing and evaluating step is O(2N+N_MKE-TVP_D+N_BTVP_D+N_RTVP_D). Because N = N_MKE-TVP+_N_BTVP_+N_RTVP_, the complexity of the while-loop (lines 7–26) is O(2N+ND). The cost of creating X^BHpop^ (line 24) is O(N), then the evolution’s complexity for all generations (G) is O(ND+G(2N+ND)). The overall computational complexity of the MMKE algorithm is equivalent to O(ND+2GN+GND) or O(GND).

## 5. Experimental evaluation and results

Various experiments were designed to evaluate our proposed algorithm’s performance by using different problems of the CEC 2018 [27]. First, a visual analysis of MMKE is provided along with two directions: trial vector impact analysis and exploration and exploitation behavior analysis. These experiments aimed to demonstrate the impact, convergence behavior, and explorative and exploitative tendencies of proposed trial vectors during the search process. Second, MMKE’s performance was evaluated using a quantitative analysis including exploration and exploitation, local optima avoidance, and convergence evaluation in comparison to eight state-of-the-art metaheuristic algorithms.

### 5.1 Benchmark test functions and experimental environment

As part of this study, 29 benchmark functions from the CEC 2018 test suite [27] are used in order to evaluate the proposed MMKE algorithm. There are four kinds of functions in this test suite: unimodal, simple multimodal, hybrid, and composition functions. It is imperative to analyze the algorithm’s exploitative capacity as well as its convergence behavior in issues where there is only one optimal solution. Unimodal functions (Func 1, Func 3) particularly serve this purpose. In addition, it is possible to test the algorithm’s exploratory and local optima avoidance abilities using multimodal functions (Func 4-Func 10) having more than one local optimum. Considering the importance of striking a balance between exploration and exploitation when solving real-world issues, hybrid (Func 11–Func 20) and composition (Func 21–Func 30) functions are suitable for benchmarking this capability. The MMKE was developed by Matlab programming environment R2018a, and all experiments were run using an Intel i7 CPU with 3.4GHz and 8.00 GB memory.

### 5.2 Visual analysis

In this experiment set, the MMKE’s visual analysis was performed on a number of selected functions of the CEC 2018 to analyse the impact of proposed trial vectors and exploration and exploitation behavior. First, to analyse the impact of introduced TVPs in the MMKE algorithm, the convergence of each TVP and the improved rate of each TVP is performed. Then, the exploration and exploitation tendency of MMKE is shown. All analyses were performed over *(Dim×10000)/N* generations, where *N* is the population size that set to 100 and *Dim* is the dimensions of the problem, which varies of 10, 30, and 50.

#### 5.2.1 Trial vector impact analysis

**Fig 9:**
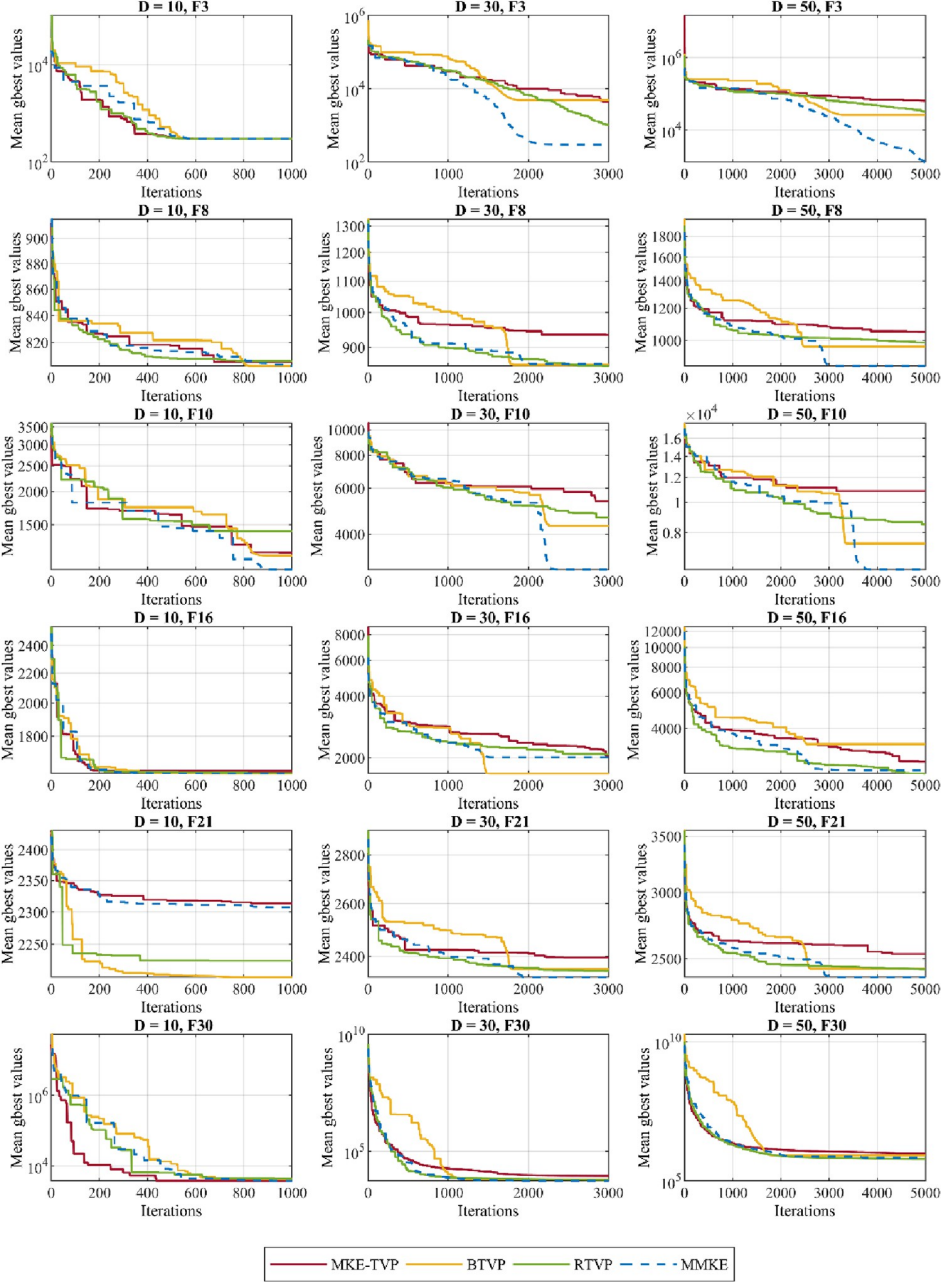
The TVPs’ impact analysis.

**Fig 10:**
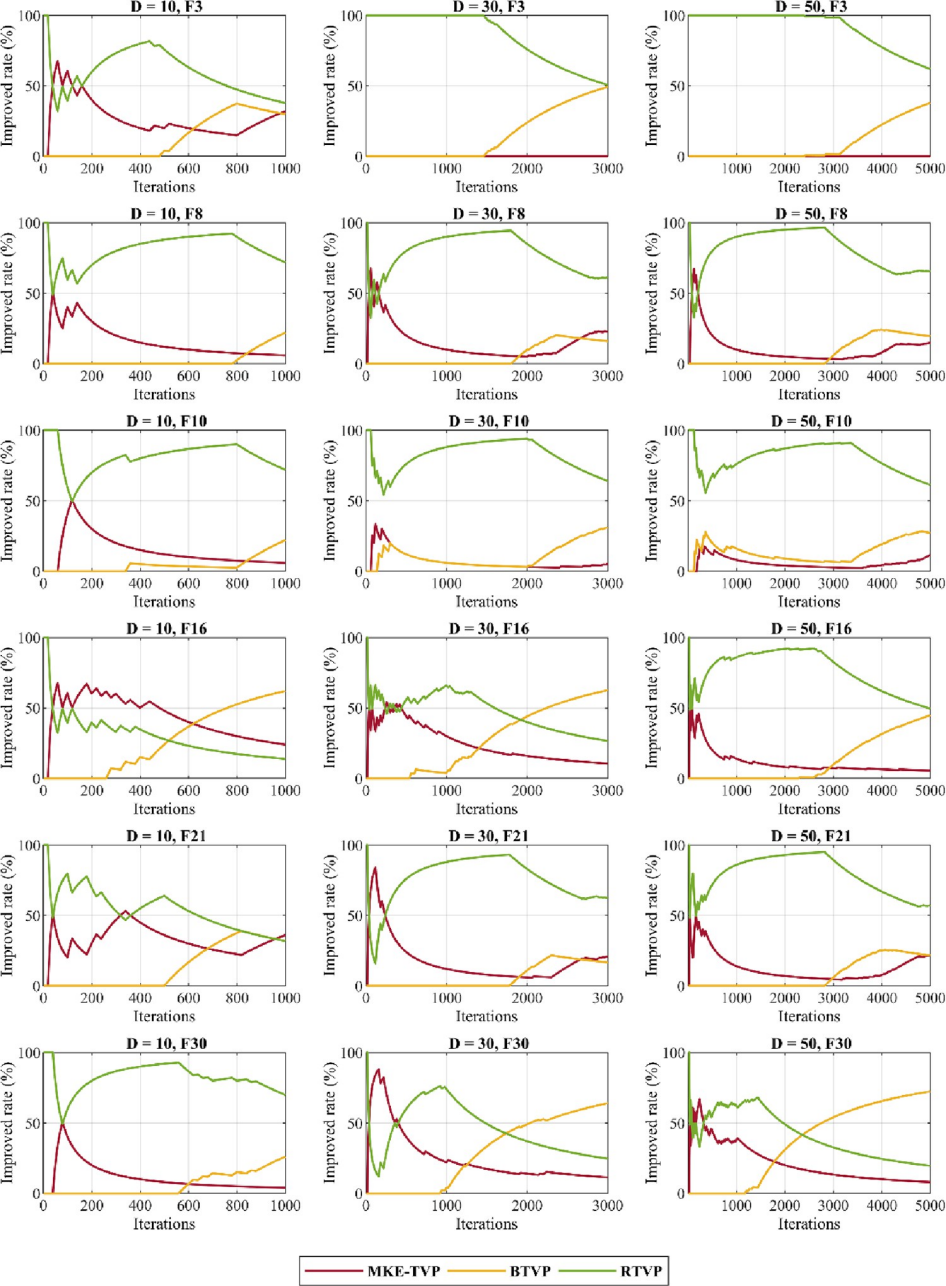
The TVPs’ improved rate in percentage.

In the following subsection, the impact of the BTVP and RTVP evolution strategies examine on the performance of MMKE using two separate tests. The curves of these tests are shown in Figs 9 and 10 on six functions Func 3, Func 8, Func 10, Func 16, Func 21, and Func 30. In the first test, each MKE-TVP, BTV, and RTVP was considered as a distinct algorithm, and the best obtained result in each generation was compared to MMKE. As the curves shown in Fig 9, in comparison to MKE-TVP, better solutions are obtained by BTVP in unimodal, simple multimodal, hybrid, and composition functions. Thus, the findings of this analysis reveal that by using gbest-history, the BTVP is able to avoid premature convergence and entrapment in local optima, as well as perform superior exploitation. The RTVP is also able to better find optimal solutions for the hybrid and composition functions that can adjust a balance between exploration and exploitation and avoid premature convergence. This TVP has a top priority for exploring and leading the search to the global optimum. Differences between random solutions are utilized to balance exploration and exploitation and preserve diversity throughout the optimization.

In the second test, the improved rate of each TVP in the MMKE algorithm is calculated, and the percent of their improvement is indicated on the aforementioned functions of CEC 2018. As the curves shown in Fig 10, it is evident that BTVP and RTVP are dominant evolution strategies that have unique effects on the search process, while MKE-TVP has the least impact on the optimization process. Also, the improved rate of each TVP differs in functions with various features or even in different phases of the search process. By considering the curves shown in this figure, it reveals that the combination of MKE-TVP and BTVP has a superior effect on dealing with unimodal problems, while the cooperation of BTVP and RTVP in solving complex problems with many local optima is most significant.

#### 5.2.2 Exploration and exploitation analysis

In this section, the explorative and exploitative capabilities of the MMKE algorithm are examined on selected functions of CEC 2018. The exploration ability of an algorithm is to globally investigate more regions of the search space, while exploitation refers to locally search potential solutions in the promising regions to increase the efficiency of the found solution. Since metaheuristic algorithms use a population of solutions, the effect of exploration is more apparent when the distance between the solutions increases. On the other hand, the effect of exploitation increases when the distance among the solutions decreases. Depending on the search strategy of an algorithm, a tradeoff between exploration and exploitation is needed to achieve a reasonable solution. To analyze this behavior, a dimension-wise diversity measurement [107] shown in Eq (13) is used to measure the distance among the solutions.


$${Div}_{}=\frac{1}{D}\sum_{j=1}^{D}\frac{1}{N}\sum_{i=1}^{N}\left|median(x_{j}(t)-x_{ij}(t)\right|$$ (13)

**Fig 11:**
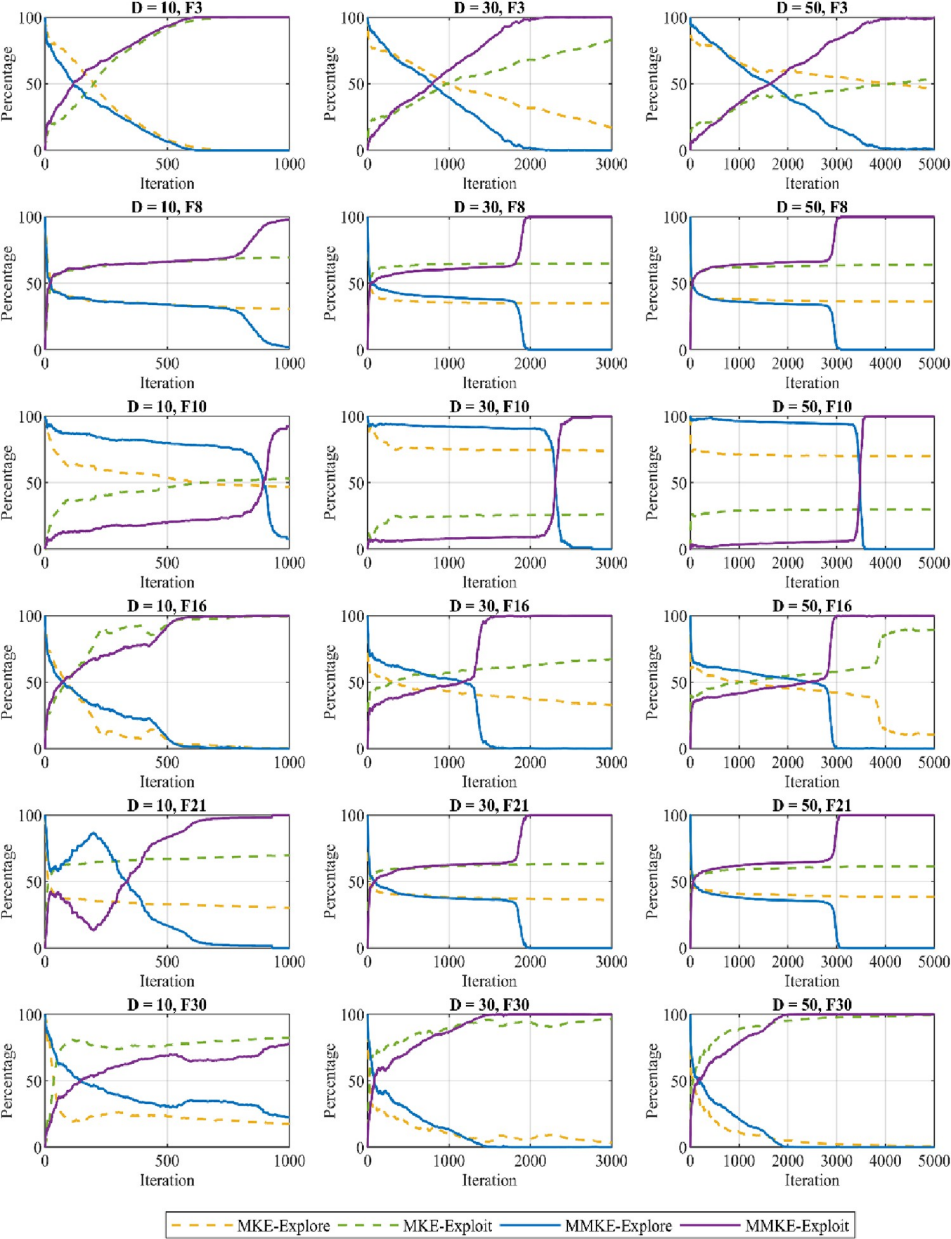
The balance of exploration and exploitation in MMKE.

Where Div is the diversity measurement of the whole population in each generation, Dim and N are dimensions and the size of the population, and median x_j_(t) refers to the median of dimension j in the whole population. The exploration and exploitation invested by an algorithm can be calculated using Eq (14) and Eq (15),

$$XPL\%=\frac{Div}{{Div}_{max}}\times 100$$ (14)

$$XPT\%=\frac{\left|Div-{Div}_{max}\right|}{{Div}_{max}}\times 100$$ (15)

where Div_max_ is the maximum diversity value during the optimization process. As can be observed from the plotted curves shown in Fig 11, both XPL% and XPT% are mutually complement each other and MMKE can be achieved an adequate balance between exploration and exploitation during the search process. Also, the figures demonstrate that MMKE starts to explore the search space through multiple candidate solutions. Then, exploration behavior is transformed into exploitation depending on the problem to be solved by a smooth transition.

The plotted curves for the unimodal test functions Func 1 are shown in Fig 11 for dimensions 10, 30, and 50, which indicate that individuals investigate search space with a high proportion of exploration during the first one-fourth of the evolutionary process. Then, individuals gradually modify their behavior to accelerate convergence to a global optimal with a large proportion of exploitation. The exploration capability of the proposed algorithm was evaluated using multimodal test functions Func 8 and Func 10, including several local optima. The plotted curves for these test functions indicate that individuals alternate between exploration and exploitation to decrease the probability of being trapped in local optima. In addition, the large proportion of exploration signifies that individuals tend to enhance the possibility of discovering new areas inside the search space, although exploiting the optimal solution is continued in the last iterations. The plotted curves depicted for Func 16, Func 21, and Func 30 show that individuals explore the search space with a large proportion of exploration during the initial iterations, and this ratio subsequently decreases until this behavior transitions into the exploitation capacity, which grows to accelerate convergence speed.

### 5.3 Quantitative evaluation

Analyzing the performance of the MMKE algorithm is the objective of this subsection, which includes an extensive experimental study and a statistical evaluation. On the basis of the gained results, a comparison is conducted with state-of-the-art algorithms, including grey wolf optimizer (GWO) [17], whale optimization algorithm (WOA) [4], salp swarm algorithm (SSA) [14], butterfly optimization algorithm (BOA) [108], and Aquila optimizer (AO) [109] from swarm intelligence algorithms and well-known composite DE (CoDE) [94], the ensemble of mutation strategies and parameters in DE (EPSDE) [100], quasi-affine transformation evolutionary (QUATRE) [110], and monkey king evolution (MKE) [25] from the same category of evolutionary algorithms.

The values of any parameters related to the comparative algorithms were set in accordance with the recommendations from the original article, as shown in Table 2. A total of 20 separate runs with varying dimensions of 10, 30, and 50 were used to assess all benchmark functions. Every time a run was performed, the maximum number of generations (*MaxGen*) was determined by *(Dim×10000)/N*, where *Dim* and *N* were set to the problem’s dimensions and a constant of 100. Reporting the obtained results is done by using the fitness error value f(*gbest*^*pop*^)–f(X*), where f(*gbest*^*pop*^) signifies the minimum fitness value gained and f(X*) denotes the actual global optimum solution of the test function. The mean and standard deviation of the error values were used to assess the algorithms’ performance. The experimental results are shown in Tables 3–6, in which the best-obtained error values are remarked in boldface. Furthermore, the bottom three consecutive rows of each table designated "l/t/w" represent the number of algorithm losses (l), ties (t), and wins (w).

**Table 2:** The parameter settings of algorithms.

Algorithms	Parameters values
GWO, WOA	*a* = [2 0]
SSA	*c*_*2*_, *c*_*3*_ = random numbers in [0, 1]
AO	*α = δ = 0*.*1*
CoDE	*CR* = [0.1, 0.9, 0.2], *F* = [1, 1, 0.8]
EPSDE	*CR* = [0.1, 0.9], *F* = [0.4, 0.9]
QUATRE	*FC* = 0.7
MMKE	*ngen* = 20, *M* = 5, *α* = 0.001, *β* = 2, *μ* = log(*Dim*)

**Table 3:** The obtained results for unimodal test functions.

Func #	Dim	Metric	GWO	WOA	SSA	BOA	AO	CoDE	EPSDE	QUATRE	MKE	MMKE
Func 1	10	Avg	2.6936E+06	2.3700E+05	3.1749E+03	9.7702E+09	2.2624E+05	1.2755E+00	**0.0000E+00**	**0.0000E+00**	**0.0000E+00**	**0.0000E+00**
		SD	7.2072E+06	4.3642E+05	2.9583E+03	2.3917E+09	1.4225E+05	6.8568E-01	0.0000E+00	0.0000E+00	0.0000E+00	0.0000E+00
	30	Avg	8.1476E+08	3.0083E+06	4.8644E+03	5.6174E+10	3.1847E+06	3.1323E+01	**0.0000E+00**	**0.0000E+00**	**0.0000E+00**	**0.0000E+00**
		SD	6.2356E+08	2.2408E+06	5.7586E+03	5.0365E+09	1.1160E+06	1.0292E+01	0.0000E+00	0.0000E+00	0.0000E+00	0.0000E+00
	50	Avg	4.7363E+09	1.0154E+07	6.7143E+03	1.0737E+11	1.1735E+07	2.7340E+03	**0.0000E+00**	2.3615E-04	3.2384E-02	1.0573E-04
		SD	2.1066E+09	1.0566E+07	8.5797E+03	5.1881E+09	2.5192E+06	2.5877E+03	0.0000E+00	5.1790E-04	4.4004E-02	1.3220E-04
Func 3	10	Avg	5.3309E+02	1.6779E+02	1.1679E-09	1.3524E+04	1.1512E+01	1.7841E-02	**0.0000E+00**	**0.0000E+00**	1.3158E-06	**0.0000E+00**
		SD	8.2463E+02	1.5222E+02	4.4346E-10	2.8594E+03	1.2833E+01	1.5514E-02	0.0000E+00	0.0000E+00	1.7859E-06	0.0000E+00
	30	Avg	2.6220E+04	1.5432E+05	**3.6089E-08**	8.3024E+04	6.0179E+03	1.0315E+00	1.4991E+01	7.6683E-04	6.9121E+03	5.5310E-03
		SD	7.0614E+03	7.2660E+04	1.1043E-08	8.1575E+03	1.5948E+03	5.7046E-01	6.5823E+01	1.3767E-03	2.0852E+03	6.4540E-03
	50	Avg	6.7629E+04	6.9827E+04	**1.9602E-07**	2.3521E+05	5.4669E+04	9.2546E+00	2.6866E+05	4.5795E+01	5.6432E+04	1.2089E+03
		SD	1.5462E+04	2.7736E+04	5.4646E-08	5.7252E+04	6.9606E+03	5.8621E+00	2.2933E+05	3.0281E+01	1.0679E+04	4.1105E+02
**Rank**	**10**	**(l/t/w)**	2/0/0	2/0/0	2/0/0	2/0/0	0/2/0	2/0/0	0/2/0	0/2/0	1/1/0	0/2/0
	**30**	**(l/t/w)**	2/0/0	2/0/0	1/0/1	2/0/0	0/2/0	2/0/0	1/1/0	1/1/0	1/1/0	1/1/0
	**50**	**(l/t/w)**	2/0/0	2/0/0	1/0/1	2/0/0	0/2/0	2/0/0	1/0/1	2/0/0	2/0/0	2/0/0

**Table 4:** The obtained results for simple multimodal test functions.

Func #	Dim	Metric	GWO	WOA	SSA	BOA	AO	CoDE	EPSDE	QUATRE	MKE	MMKE
Func 4	10	Avg	7.8487E+00	4.0139E+01	3.9742E+00	1.2370E+03	5.3668E+00	5.1561E-03	**0.0000E+00**	**0.0000E+00**	1.6905E-02	4.2814E-02
		SD	2.7641E+00	4.5178E+01	1.5470E+00	3.2145E+02	2.7193E+00	1.8530E-03	0.0000E+00	0.0000E+00	1.2835E-02	1.2965E-02
	30	Avg	1.5949E+02	1.5829E+02	8.7145E+01	2.1165E+04	1.2033E+02	8.1949E+01	**1.9933E-01**	5.1643E+01	2.7683E+01	2.0426E+01
		SD	4.1578E+01	3.9381E+01	1.7130E+01	3.6209E+03	4.4275E+01	5.4621E+00	8.9144E-01	2.1843E+01	3.0449E+01	2.1501E+01
	50	Avg	4.4232E+02	2.8183E+02	1.4073E+02	4.1621E+04	2.1898E+02	5.7050E+01	**1.2897E+01**	6.8273E+01	8.6013E+01	7.2119E+01
		SD	1.8550E+02	6.4627E+01	5.0752E+01	5.2965E+03	5.8845E+01	4.1001E+01	1.4051E+01	5.1426E+01	5.5424E+01	3.7261E+01
Func 5	10	Avg	1.1370E+01	4.4390E+01	1.8456E+01	1.1849E+02	2.7027E+01	1.3083E+01	4.6813E+00	9.7378E+00	1.0651E+01	**2.6366E+00**
		SD	4.4095E+00	2.2590E+01	9.2858E+00	1.3523E+01	9.6599E+00	2.4619E+00	1.2234E+00	4.2841E+00	2.2667E+00	7.4140E-01
	30	Avg	9.5317E+01	2.6401E+02	1.2732E+02	5.8562E+02	1.5762E+02	1.3589E+02	5.5854E+01	6.6602E+01	1.0981E+02	**2.8307E+01**
		SD	2.0311E+01	5.5593E+01	2.9601E+01	4.6640E+01	2.9011E+01	1.0157E+01	5.3044E+00	1.7212E+01	1.0068E+01	5.7517E+00
	50	Avg	1.8147E+02	4.1296E+02	3.0417E+02	9.5053E+02	2.9415E+02	2.9638E+02	1.7334E+02	1.4851E+02	2.4883E+02	**7.1090E+01**
		SD	3.4472E+01	8.0627E+01	6.4642E+01	5.1820E+01	4.1151E+01	2.0240E+01	1.5788E+01	2.9009E+01	1.4592E+01	7.4559E+00
Func 6	10	Avg	1.1894E+00	3.1527E+01	6.3763E+00	6.5093E+01	9.3669E+00	1.5789E-03	5.6843E-15	6.7772E-07	8.5265E-14	**0.0000E+00**
		SD	1.5200E+00	1.2828E+01	7.5525E+00	1.3798E+01	3.8987E+00	4.1862E-04	2.5421E-14	2.7150E-06	5.0507E-14	0.0000E+00
	30	Avg	5.2351E+00	6.9296E+01	3.1236E+01	1.1713E+02	3.7565E+01	3.5183E-02	**0.0000E+00**	4.8730E-06	6.1419E-08	3.2745E-05
		SD	2.8082E+00	9.7291E+00	1.3201E+01	1.2387E+01	5.2719E+00	5.8565E-03	0.0000E+00	6.6260E-06	2.4437E-07	1.6826E-05
	50	Avg	1.1030E+01	7.5389E+01	4.3445E+01	1.3207E+02	5.2480E+01	2.2104E-02	**0.0000E+00**	5.2786E-02	8.9129E-07	1.1673E-04
		SD	3.9609E+00	9.2975E+00	9.4786E+00	7.7612E+00	6.1813E+00	5.1171E-03	0.0000E+00	1.6546E-01	2.0783E-06	4.7099E-05
Func 7	10	Avg	2.5162E+01	7.6079E+01	3.4645E+01	3.2142E+02	4.7231E+01	2.8806E+01	1.6181E+01	1.8731E+01	2.2055E+01	**1.3318E+01**
		SD	8.5271E+00	3.2575E+01	1.2508E+01	6.5306E+01	1.3861E+01	3.6728E+00	1.4943E+00	4.7393E+00	2.8185E+00	1.1527E+00
	30	Avg	1.5280E+02	5.0438E+02	1.6410E+02	1.1549E+03	2.7505E+02	1.7836E+02	9.0265E+01	1.0289E+02	1.4935E+02	**6.2925E+01**
		SD	4.9621E+01	1.0117E+02	3.0913E+01	1.1713E+02	4.0861E+01	9.0470E+00	7.3344E+00	1.3817E+01	1.3173E+01	4.4816E+00
	50	Avg	3.3824E+02	1.0075E+03	3.3601E+02	1.8620E+03	5.7082E+02	3.6599E+02	2.2897E+02	2.1822E+02	3.1569E+02	**1.1086E+02**
		SD	8.3081E+01	1.0137E+02	9.2109E+01	1.2774E+02	9.0809E+01	1.4569E+01	1.2009E+01	3.2075E+01	1.4007E+01	8.1794E+00
Func 8	10	Avg	1.0379E+01	4.0549E+01	1.7263E+01	1.0390E+02	2.2191E+01	1.4827E+01	5.5220E+00	9.6122E+00	1.1319E+01	**3.0362E+00**
		SD	3.6736E+00	1.5843E+01	9.3740E+00	1.3239E+01	6.6420E+00	2.6524E+00	1.4668E+00	4.0365E+00	2.2883E+00	6.8279E-01
	30	Avg	7.7273E+01	2.1580E+02	1.1934E+02	4.8186E+02	1.3289E+02	1.3824E+02	5.7414E+01	7.1570E+01	1.1418E+02	**3.0452E+01**
		SD	2.8266E+01	5.5436E+01	2.7226E+01	3.6216E+01	2.7277E+01	6.3473E+00	5.7066E+00	1.6854E+01	8.2390E+00	4.6568E+00
	50	Avg	2.0225E+02	4.1131E+02	2.8890E+02	9.9076E+02	3.0518E+02	2.9429E+02	1.7693E+02	1.6358E+02	2.5042E+02	**6.5928E+01**
		SD	3.1759E+01	7.2877E+01	8.1083E+01	5.4246E+01	5.3405E+01	2.0582E+01	1.4681E+01	3.6884E+01	1.5163E+01	8.8744E+00
Func 9	10	Avg	6.1717E+00	4.5187E+02	4.7899E+00	2.5353E+03	6.2282E+01	2.0539E-05	**0.0000E+00**	**0.0000E+00**	5.6843E-15	**0.0000E+00**
		SD	1.5826E+01	3.3710E+02	1.8037E+01	6.6907E+02	4.6892E+01	2.5090E-05	0.0000E+00	0.0000E+00	2.5421E-14	0.0000E+00
	30	Avg	5.2873E+02	6.4165E+03	2.2272E+03	2.6869E+04	3.3454E+03	5.3077E-01	3.2330E-03	5.8614E-01	9.9818E-02	**0.0000E+00**
		SD	3.6007E+02	2.0378E+03	1.2425E+03	4.9770E+03	8.7093E+02	2.3929E-01	1.4451E-02	1.4372E+00	1.8388E-01	0.0000E+00
	50	Avg	4.4885E+03	2.0131E+04	1.0124E+04	7.9929E+04	1.3028E+04	6.1739E-02	9.9732E-01	9.7230E+01	4.6261E-01	**0.0000E+00**
		SD	3.2635E+03	6.7791E+03	2.2154E+03	5.9504E+03	2.9579E+03	9.8630E-02	2.8696E+00	3.3040E+02	5.5392E-01	0.0000E+00
Func 10	10	Avg	5.2074E+02	9.4125E+02	9.1517E+02	2.2427E+03	6.9539E+02	5.9908E+02	4.0650E+02	3.3879E+02	4.8703E+02	**1.3434E+01**
		SD	3.1319E+02	2.4674E+02	2.5987E+02	2.1553E+02	3.0102E+02	1.0306E+02	1.0178E+02	1.6816E+02	1.4692E+02	8.9351E+00
	30	Avg	3.2464E+03	5.1831E+03	3.5990E+03	8.9329E+03	3.8183E+03	5.0346E+03	4.2168E+03	3.1226E+03	4.4796E+03	**2.0002E+03**
		SD	9.3032E+02	7.2715E+02	5.5099E+02	3.1834E+02	6.1713E+02	3.0123E+02	3.2407E+02	4.4122E+02	4.2501E+02	2.2204E+02
	50	Avg	5.4515E+03	8.8691E+03	6.4743E+03	1.5646E+04	6.5191E+03	1.0192E+04	9.1234E+03	7.1913E+03	9.3447E+03	**4.3089E+03**
		SD	7.8058E+02	1.1283E+03	1.0281E+03	4.3295E+02	9.8610E+02	3.2791E+02	4.5276E+02	8.2756E+02	3.4200E+02	4.9506E+02
**Rank**	**10**	**(l/t/w)**	7/0/0	7/0/0	7/0/0	7/0/0	7/0/0	7/0/0	5/2/0	5/2/0	7/0/0	1/1/5
	**30**	**(l/t/w)**	7/0/0	7/0/0	7/0/0	7/0/0	7/0/0	7/0/0	5/0/2	7/0/0	7/0/0	2/0/5
	**50**	**(l/t/w)**	7/0/0	7/0/0	7/0/0	7/0/0	7/0/0	7/0/0	5/0/2	7/0/0	7/0/0	2/0/5

**Table 5:** The obtained results for hybrid test functions.

Func #	Dim	Metric	GWO	WOA	SSA	BOA	AO	CoDE	EPSDE	QUATRE	MKE	MMKE
Func 11	10	Avg	1.9701E+01	8.5048E+01	9.4305E+01	1.6971E+03	6.5036E+01	4.5977E+00	2.6263E+00	2.7222E+00	7.3230E-01	**9.9496E-02**
		SD	9.5902E+00	5.7137E+01	7.9869E+01	1.3442E+03	2.8446E+01	8.5711E-01	7.4468E-01	1.9369E+00	1.1053E+00	3.0624E-01
	30	Avg	2.3004E+02	3.5236E+02	1.6649E+02	6.9994E+03	2.3845E+02	6.7356E+01	2.2598E+01	4.0313E+01	5.1794E+01	**7.0905E+00**
		SD	4.8676E+01	9.7563E+01	4.7438E+01	1.9350E+03	6.5398E+01	2.0836E+01	5.9317E+00	2.9007E+01	3.8859E+01	2.2540E+00
	50	Avg	1.3424E+03	4.6940E+02	2.7692E+02	2.4474E+04	3.5228E+02	9.5524E+01	8.4989E+01	1.3917E+02	1.1583E+02	**4.5005E+01**
		SD	1.0928E+03	1.0374E+02	7.4539E+01	2.9482E+03	6.9666E+01	2.4815E+01	4.4216E+01	5.4759E+01	3.5544E+01	6.8801E+00
Func 12	10	Avg	4.8726E+05	4.2120E+06	1.0344E+06	4.1210E+08	2.7730E+06	2.8640E+02	1.0908E+02	1.8454E+02	2.9447E+02	**7.9903E+01**
		SD	7.1855E+05	5.9617E+06	1.6575E+06	1.8812E+08	2.4568E+06	6.3462E+01	1.5482E+02	1.7062E+02	1.6237E+02	6.7506E+01
	30	Avg	3.3989E+07	3.6939E+07	2.2560E+06	1.5487E+10	1.2367E+07	**2.5892E+03**	4.5615E+04	1.5134E+04	1.5506E+04	5.7579E+03
		SD	3.9251E+07	2.8031E+07	2.5525E+06	2.6744E+09	5.3106E+06	1.1808E+03	4.4471E+04	1.4375E+04	1.1730E+04	3.7950E+03
	50	Avg	4.6164E+08	1.8458E+08	1.8689E+07	8.3041E+10	5.3329E+07	9.2217E+04	2.2565E+05	6.5904E+04	9.3254E+04	**3.0629E+04**
		SD	6.1781E+08	9.2767E+07	1.1690E+07	9.3296E+09	3.5845E+07	6.0451E+04	1.3454E+05	4.2030E+04	4.4857E+04	1.3071E+04
Func 13	10	Avg	7.9340E+03	1.5234E+04	1.2144E+04	4.7861E+06	1.1589E+04	1.1016E+01	6.6574E+00	6.9931E+00	5.9751E+00	**3.0267E+00**
		SD	6.7034E+03	1.0382E+04	1.0960E+04	5.6843E+06	8.0174E+03	1.9231E+00	1.2419E+00	2.7752E+00	4.1283E+00	2.0191E+00
	30	Avg	1.0140E+07	1.2115E+05	1.1858E+05	1.3810E+10	2.3444E+05	1.0519E+02	2.1091E+02	2.4022E+02	1.1235E+02	**3.1343E+01**
		SD	3.0220E+07	6.2436E+04	7.8704E+04	3.4469E+09	1.2381E+05	8.9029E+00	3.9523E+02	4.5269E+02	4.5668E+01	7.3672E+00
	50	Avg	7.9170E+07	1.5436E+05	1.1479E+05	5.0392E+10	5.7355E+05	**2.4947E+02**	5.0622E+03	1.5081E+03	5.8228E+03	1.1284E+03
		SD	1.0185E+08	7.4862E+04	6.1017E+04	1.0450E+10	2.1883E+05	3.1585E+01	8.6389E+03	5.5496E+03	7.0074E+03	9.9250E+02
Func 14	10	Avg	9.2984E+02	3.3810E+02	8.5938E+01	4.1676E+03	3.5378E+02	5.6295E+00	2.0179E+01	1.2591E+01	6.7102E-01	**4.4773E-01**
		SD	1.5298E+03	6.4869E+02	2.7485E+01	4.7082E+03	3.7947E+02	1.1535E+00	1.4918E+00	9.8647E+00	6.1418E-01	5.0784E-01
	30	Avg	1.0906E+05	9.5530E+05	5.6744E+03	4.9894E+06	9.4088E+04	5.1292E+01	4.3521E+01	8.2280E+01	5.0334E+01	**2.9373E+01**
		SD	2.0586E+05	1.5190E+06	4.5919E+03	2.6076E+06	8.2181E+04	5.3422E+00	9.4334E+00	4.5761E+01	1.0133E+01	2.7682E+00
	50	Avg	4.5484E+05	5.5205E+05	5.2942E+04	1.5267E+08	6.5814E+05	9.4391E+01	2.5571E+02	2.2464E+02	1.2188E+02	**4.6395E+01**
		SD	7.1116E+05	4.0055E+05	3.8724E+04	8.7508E+07	4.3006E+05	1.0579E+01	1.4043E+02	1.4114E+02	1.7404E+01	7.1188E+00
Func 15	10	Avg	1.3381E+03	2.3648E+03	5.2345E+02	1.8893E+04	1.8253E+03	1.7661E+00	5.4421E-01	1.0820E+00	4.0578E-01	**4.6232E-02**
		SD	1.5180E+03	2.7828E+03	5.3881E+02	1.1977E+04	1.0919E+03	3.8889E-01	4.5094E-01	7.4875E-01	5.6612E-01	5.7034E-02
	30	Avg	1.3431E+05	8.8391E+04	4.5520E+04	1.1021E+09	6.6141E+04	3.6380E+01	6.3875E+01	5.2284E+01	4.0541E+01	**8.2638E+00**
		SD	4.5283E+05	4.7060E+04	2.8470E+04	3.6327E+08	5.1010E+04	4.0614E+00	5.9824E+01	4.7524E+01	2.7569E+01	2.5450E+00
	50	Avg	4.8137E+06	7.7435E+04	4.4971E+04	9.5803E+09	1.5026E+05	8.0859E+01	2.0920E+02	2.1065E+02	1.1593E+02	**5.0609E+01**
		SD	8.7240E+06	6.3161E+04	3.0186E+04	2.4727E+09	5.7380E+04	1.1678E+01	1.9079E+02	9.7264E+01	2.0690E+01	9.8997E+00
Func 16	10	Avg	8.0516E+01	1.7716E+02	9.0012E+01	7.3601E+02	1.3963E+02	4.3984E+00	1.5174E+01	5.4826E+01	7.4553E+00	**3.2658E-01**
		SD	7.5621E+01	1.2895E+02	9.3476E+01	1.7751E+02	1.1097E+02	1.2698E+00	6.2396E+00	8.0120E+01	2.6358E+01	1.2715E-01
	30	Avg	6.5336E+02	1.8852E+03	9.8454E+02	4.9447E+03	1.2135E+03	7.1072E+02	6.7917E+02	8.4932E+02	4.3748E+02	**2.5417E+02**
		SD	2.8241E+02	4.9031E+02	2.3543E+02	7.2397E+02	3.5766E+02	1.0700E+02	1.6662E+02	2.6359E+02	2.7036E+02	1.3060E+02
	50	Avg	1.2954E+03	3.0592E+03	1.5807E+03	9.8435E+03	2.1509E+03	1.5917E+03	1.3719E+03	1.3897E+03	1.2226E+03	**7.7220E+02**
		SD	3.0457E+02	7.2440E+02	4.2003E+02	1.3719E+03	4.6546E+02	3.3049E+02	1.9557E+02	3.2689E+02	4.4406E+02	2.0267E+02
Func 17	10	Avg	5.3811E+01	9.7431E+01	5.5165E+01	3.3417E+02	5.8279E+01	1.7364E+01	2.0831E+01	2.6272E+01	8.6833E+00	**3.7087E-01**
		SD	3.0730E+01	6.1952E+01	1.6261E+01	1.0912E+02	1.7090E+01	2.1923E+00	5.3884E+00	3.5899E+01	1.7414E+01	2.2703E-01
	30	Avg	2.5303E+02	7.2939E+02	3.5041E+02	3.8562E+03	5.4176E+02	1.4952E+02	2.4680E+02	1.4841E+02	1.3210E+02	**4.0423E+01**
		SD	1.3331E+02	2.1697E+02	1.6716E+02	1.7286E+03	1.6947E+02	3.2047E+01	8.0924E+01	1.1734E+02	5.7431E+01	8.0561E+00
	50	Avg	9.8963E+02	2.3482E+03	1.4008E+03	1.7082E+04	1.6435E+03	1.0202E+03	9.5925E+02	9.8996E+02	8.1060E+02	**4.5664E+02**
		SD	2.5497E+02	3.4275E+02	2.3268E+02	8.0980E+03	3.6818E+02	1.5162E+02	1.2572E+02	2.3941E+02	2.4805E+02	9.7341E+01
Func 18	10	Avg	2.3830E+04	9.1182E+03	2.1367E+04	6.4501E+07	2.4003E+04	5.5952E+00	1.9351E+01	1.7835E+01	4.7390E+00	**1.6141E-01**
		SD	1.6466E+04	8.0976E+03	1.4669E+04	7.1919E+07	1.3873E+04	8.8690E-01	2.9851E+00	1.2328E+01	7.9763E+00	1.6100E-01
	30	Avg	6.7335E+05	1.9281E+06	1.5707E+05	1.0521E+08	1.0231E+06	**4.1279E+01**	2.6412E+02	6.4504E+01	6.6905E+01	4.4423E+01
		SD	8.4951E+05	2.1247E+06	1.5509E+05	7.3852E+07	7.2859E+05	3.6041E+00	2.6325E+02	3.2442E+01	3.2365E+01	1.0211E+01
	50	Avg	3.0602E+06	4.9346E+06	4.1591E+05	2.4993E+08	3.7985E+06	**6.9730E+01**	2.8082E+03	4.0956E+03	3.1495E+03	1.2223E+03
		SD	4.0639E+06	5.1695E+06	2.1358E+05	9.0045E+07	2.1464E+06	1.5608E+01	2.5804E+03	3.8959E+03	1.9190E+03	5.4361E+02
Func 19	10	Avg	4.1600E+03	1.2711E+04	8.0869E+02	1.0653E+05	3.2745E+03	1.0741E+00	8.1032E-01	1.5541E+00	3.8454E-01	**1.6517E-02**
		SD	5.5781E+03	1.1561E+04	1.6812E+03	1.1615E+05	3.1393E+03	2.8534E-01	3.1367E-01	1.0883E+00	7.2222E-01	7.1188E-03
	30	Avg	3.5439E+05	2.6507E+06	3.9762E+05	1.0527E+09	3.9096E+05	2.6320E+01	1.9979E+01	3.0166E+01	1.3136E+01	**9.6118E+00**
		SD	4.6865E+05	2.7677E+06	2.5112E+05	4.9151E+08	3.0493E+05	3.2389E+00	3.8950E+00	2.0669E+01	3.9191E+00	1.4389E+00
	50	Avg	3.2081E+06	2.3104E+06	6.5237E+05	4.7424E+09	6.1083E+05	4.8120E+01	6.1557E+01	1.0128E+02	4.8194E+01	**2.9314E+01**
		SD	7.3057E+06	2.0747E+06	3.5169E+05	1.3844E+09	4.1220E+05	6.8592E+00	3.5392E+01	3.8509E+01	1.9089E+01	2.7358E+00
Func 20	10	Avg	7.6925E+01	1.3176E+02	7.3270E+01	2.7479E+02	7.3709E+01	4.3993E-01	1.6571E+01	8.0695E+00	6.0618E+00	**1.2487E-01**
		SD	6.1548E+01	5.7658E+01	4.7158E+01	8.1726E+01	2.7974E+01	1.8870E-01	5.7182E+00	9.8664E+00	1.0350E+01	1.5691E-01
	30	Avg	4.1912E+02	6.7155E+02	4.8377E+02	1.4074E+03	4.1028E+02	1.4331E+02	1.5420E+02	2.5595E+02	1.1070E+02	**5.3814E+01**
		SD	1.3238E+02	1.8164E+02	1.3994E+02	1.5378E+02	1.1620E+02	4.9126E+01	5.9122E+01	1.6851E+02	8.5737E+01	4.9171E+01
	50	Avg	7.5885E+02	1.6766E+03	9.9999E+02	2.8807E+03	1.0341E+03	6.1653E+02	7.0643E+02	8.6896E+02	6.9616E+02	**3.6463E+02**
		SD	2.0892E+02	2.5815E+02	2.7946E+02	3.7797E+02	3.5672E+02	1.9642E+02	1.6138E+02	2.8636E+02	2.4116E+02	1.4015E+02
**Rank**	**10**	**(l/t/w)**	10/0/0	10/0/0	10/0/0	10/0/0	10/0/0	10/0/0	10/0/0	10/0/0	10/0/0	10/0/0
	**30**	**(l/t/w)**	10/0/0	10/0/0	10/0/0	10/0/0	10/0/0	8/0/2	10/0/0	10/0/0	10/0/0	2/0/8
	**50**	**(l/t/w)**	10/0/0	10/0/0	10/0/0	10/0/0	10/0/0	8/0/2	10/0/0	10/0/0	10/0/0	2/0/8

**Table 6:** The obtained results for composition test functions.

Func #	Dim	Metric	GWO	WOA	SSA	BOA	AO	CoDE	EPSDE	QUATRE	MKE	MMKE
Func 21	10	Avg	1.9614E+02	2.3460E+02	1.3061E+02	2.8459E+02	1.7774E+02	1.0592E+02	1.5730E+02	1.7445E+02	1.7428E+02	**1.0000E+02**
		SD	4.1224E+01	4.8464E+01	5.0939E+01	4.9160E+01	5.9930E+01	2.6202E+01	4.8312E+01	5.6414E+01	5.5960E+01	7.3244E-12
	30	Avg	2.8060E+02	4.5188E+02	3.1560E+02	7.1831E+02	3.2528E+02	3.3301E+02	2.6052E+02	2.7140E+02	3.1199E+02	**2.2772E+02**
		SD	1.6418E+01	7.3246E+01	2.9539E+01	5.1813E+01	6.2453E+01	9.6162E+00	8.2407E+00	2.0045E+01	1.1834E+01	4.9093E+00
	50	Avg	3.7544E+02	7.4701E+02	4.3025E+02	1.2658E+03	5.1188E+02	4.9273E+02	3.8471E+02	3.5163E+02	4.5133E+02	**2.6449E+02**
		SD	3.0298E+01	9.5699E+01	4.7570E+01	6.0478E+01	5.6546E+01	1.7678E+01	1.4231E+01	3.9948E+01	1.5299E+01	7.5386E+00
Func 22	10	Avg	1.0619E+02	1.3376E+02	8.8180E+01	1.0813E+03	1.0797E+02	**4.3357E+01**	9.6913E+01	1.0182E+02	1.0128E+02	7.5014E+01
		SD	7.3900E+00	1.0088E+02	3.2657E+01	2.6322E+02	2.2633E+00	4.9441E+01	1.4082E+01	8.0659E-01	1.0036E+00	4.4435E+01
	30	Avg	2.0456E+03	3.6439E+03	1.4902E+03	8.6244E+03	1.1494E+02	**1.0000E+02**	2.9035E+03	8.9957E+02	1.2769E+03	**1.0000E+02**
		SD	1.5506E+03	2.3704E+03	1.9986E+03	7.6311E+02	9.6972E-01	9.4554E-04	2.0334E+03	1.2571E+03	2.0994E+03	0.0000E+00
	50	Avg	6.1474E+03	9.3525E+03	7.0540E+03	1.6040E+04	6.9706E+03	8.7246E+03	9.9569E+03	7.4538E+03	9.3518E+03	**3.6589E+03**
		SD	7.5856E+02	1.2469E+03	1.5776E+03	5.5055E+02	1.8424E+03	4.4359E+03	5.0803E+02	1.3538E+03	6.1993E+02	2.1541E+03
Func 23	10	Avg	3.1284E+02	3.4248E+02	3.1847E+02	4.7943E+02	3.3474E+02	3.1393E+02	3.1322E+02	3.1122E+02	3.0746E+02	**3.0261E+02**
		SD	8.4370E+00	1.3709E+01	8.6560E+00	4.8374E+01	1.3034E+01	2.0255E+00	2.1350E+00	3.7951E+00	2.7964E+00	1.8361E+00
	30	Avg	4.3072E+02	7.0212E+02	4.5263E+02	1.3711E+03	5.6898E+02	4.8025E+02	4.1174E+02	4.1037E+02	4.5145E+02	**3.7852E+02**
		SD	3.5671E+01	6.6517E+01	3.2601E+01	1.5568E+02	4.5736E+01	7.6902E+00	9.9364E+00	1.9390E+01	9.8454E+00	4.3152E+00
	50	Avg	6.0970E+02	1.3035E+03	6.5586E+02	2.6334E+03	9.1394E+02	7.2378E+02	5.8213E+02	5.8373E+02	6.7668E+02	**4.9177E+02**
		SD	3.5568E+01	1.5268E+02	5.6923E+01	1.5849E+02	7.8416E+01	1.8367E+01	1.7904E+01	3.7836E+01	2.0890E+01	9.2253E+00
Func 24	10	Avg	3.4380E+02	3.5689E+02	3.1038E+02	5.0869E+02	3.4915E+02	2.2244E+02	3.3662E+02	3.1792E+02	3.0456E+02	**2.0830E+02**
		SD	9.4946E+00	9.7844E+01	9.1119E+01	4.6854E+01	6.0042E+01	1.2565E+02	2.5896E+00	7.4611E+01	8.8197E+01	1.1453E+02
	30	Avg	5.3382E+02	7.6947E+02	5.1174E+02	1.5868E+03	6.4264E+02	5.5398E+02	4.8137E+02	4.7983E+02	5.2809E+02	**4.4564E+02**
		SD	6.8442E+01	9.0379E+01	2.5875E+01	1.9553E+02	4.9661E+01	1.1181E+01	1.1149E+01	1.5665E+01	1.1512E+01	6.6828E+00
	50	Avg	6.9049E+02	1.2697E+03	7.0900E+02	2.8707E+03	9.4342E+02	6.4575E+02	6.7698E+02	6.5057E+02	7.4601E+02	**5.5142E+02**
		SD	8.5956E+01	1.5069E+02	5.3673E+01	2.8393E+02	8.2722E+01	8.8053E+01	1.4229E+01	3.8349E+01	1.8041E+01	1.1048E+01
Func 25	10	Avg	4.3362E+02	4.4488E+02	4.2154E+02	1.0935E+03	4.3185E+02	3.9820E+02	4.2328E+02	4.2633E+02	4.1845E+02	**3.9786E+02**
		SD	2.0757E+01	2.0129E+01	2.3849E+01	1.4983E+02	2.2141E+01	1.9559E-01	2.3566E+01	2.3516E+01	2.3182E+01	1.4183E-01
	30	Avg	4.4942E+02	4.4611E+02	4.0568E+02	3.5590E+03	3.9870E+02	3.9789E+02	**3.7841E+02**	3.8674E+02	3.8632E+02	3.8662E+02
		SD	2.5934E+01	2.6972E+01	2.1988E+01	5.1242E+02	1.6156E+01	3.8017E-02	1.0756E-01	7.8332E-01	1.2497E+00	7.5171E-01
	50	Avg	8.8298E+02	6.2219E+02	5.2520E+02	1.4643E+04	6.0868E+02	4.8910E+02	**4.4755E+02**	5.2411E+02	5.0738E+02	5.0727E+02
		SD	1.8837E+02	4.5353E+01	3.8626E+01	1.4197E+03	3.7225E+01	2.5260E+01	1.8717E+01	4.1225E+01	4.0578E+01	2.1759E+01
Func 26	10	Avg	3.2437E+02	7.4395E+02	2.9461E+02	1.6321E+03	3.5388E+02	3.0000E+02	**2.8548E+02**	2.9736E+02	2.9500E+02	3.0000E+02
		SD	5.9688E+01	4.5063E+02	7.3787E+01	3.6387E+02	1.4986E+02	1.9674E-05	6.7229E+01	2.5218E+01	2.2361E+01	0.0000E+00
	30	Avg	1.7689E+03	4.9515E+03	1.5758E+03	1.0368E+04	2.4270E+03	2.1429E+03	1.3023E+03	1.6544E+03	1.8867E+03	**1.2157E+03**
		SD	2.2496E+02	1.1525E+03	1.0800E+03	9.7725E+02	1.4937E+03	4.4295E+02	7.5383E+01	1.9675E+02	3.9692E+02	2.2155E+02
	50	Avg	3.2733E+03	1.0913E+04	2.0788E+03	1.6089E+04	1.7551E+03	3.8840E+03	2.7486E+03	2.6956E+03	3.5013E+03	**1.6696E+03**
		SD	5.3469E+02	1.5122E+03	1.8790E+03	7.0608E+02	2.0666E+03	1.5757E+02	4.8340E+02	4.5215E+02	1.6075E+02	1.2198E+02
Func 27	10	Avg	3.9523E+02	4.2436E+02	3.9483E+02	5.7185E+02	3.9845E+02	**3.8760E+02**	4.0072E+02	3.9110E+02	3.9121E+02	3.8826E+02
		SD	4.0047E+00	3.5677E+01	1.5509E+01	5.5238E+01	7.7662E+00	6.5616E-01	4.4191E+01	2.7598E+00	2.7537E+00	1.1598E+00
	30	Avg	5.2954E+02	6.4006E+02	5.3278E+02	2.1219E+03	5.7485E+02	5.0346E+02	5.0001E+02	4.9970E+02	5.0289E+02	**4.9532E+02**
		SD	1.3225E+01	7.2336E+01	1.2106E+01	3.7653E+02	2.7387E+01	9.9651E+00	8.7647E-05	1.2158E+01	1.0891E+01	5.5993E+00
	50	Avg	7.9301E+02	1.4026E+03	6.9374E+02	5.1389E+03	9.5782E+02	5.1226E+02	**5.0001E+02**	5.5031E+02	5.4194E+02	5.2602E+02
		SD	7.0529E+01	2.3708E+02	7.1030E+01	5.7605E+02	1.5256E+02	1.0809E+01	7.1482E-05	2.7741E+01	2.1735E+01	1.2148E+01
Func 28	10	Avg	5.3726E+02	4.7922E+02	4.9103E+02	9.9378E+02	5.4237E+02	**3.0000E+02**	4.7603E+02	4.6990E+02	3.7655E+02	**3.0000E+02**
		SD	9.7918E+01	1.0838E+02	1.6206E+02	1.4739E+02	9.8188E+01	2.6259E-04	8.4491E+00	1.7847E+02	1.3616E+02	2.0865E-13
	30	Avg	5.3765E+02	4.9436E+02	4.0895E+02	5.3227E+03	4.7057E+02	3.3213E+02	4.9963E+02	3.3566E+02	3.3668E+02	**3.0000E+02**
		SD	6.1685E+01	2.6276E+01	3.3489E+01	7.8232E+02	3.2362E+01	3.9776E+01	1.2179E+00	5.6614E+01	5.1341E+01	1.3041E-13
	50	Avg	1.1503E+03	6.3839E+02	4.8895E+02	1.1692E+04	5.4272E+02	**4.5885E+02**	5.0001E+02	4.8430E+02	4.8014E+02	4.6779E+02
		SD	3.5418E+02	6.4159E+01	2.0504E+01	1.3846E+03	3.2914E+01	1.2604E-03	1.6171E-02	2.3796E+01	2.4333E+01	1.8447E+01
Func 29	10	Avg	2.7165E+02	4.2308E+02	2.6891E+02	6.8632E+02	3.0297E+02	2.6247E+02	2.5192E+02	2.4842E+02	2.6032E+02	**2.3557E+02**
		SD	1.7381E+01	7.6486E+01	3.0117E+01	1.3040E+02	4.7613E+01	6.0718E+00	1.0557E+01	1.4246E+01	2.9329E+01	1.3620E+00
	30	Avg	7.5712E+02	1.8765E+03	1.0873E+03	6.1277E+03	1.2297E+03	6.9229E+02	5.6641E+02	5.7772E+02	5.4841E+02	**4.6267E+02**
		SD	1.4023E+02	2.9345E+02	1.7555E+02	2.5079E+03	1.5970E+02	4.2984E+01	1.0707E+02	1.4608E+02	8.6615E+01	2.1864E+01
	50	Avg	1.3296E+03	4.0178E+03	1.8041E+03	1.3003E+05	2.3577E+03	1.0479E+03	1.0868E+03	9.0517E+02	7.0696E+02	**4.5401E+02**
		SD	2.9071E+02	7.4330E+02	2.3337E+02	8.2266E+04	4.3220E+02	1.3026E+02	1.7259E+02	3.1452E+02	2.2811E+02	4.1591E+01
Func 30	10	Avg	7.6233E+05	6.4443E+05	1.6375E+05	3.9928E+07	1.1970E+05	5.1510E+02	**2.0557E+02**	2.6717E+05	2.8711E+05	7.2482E+02
		SD	1.0643E+06	6.3156E+05	4.6598E+05	3.0313E+07	2.2524E+05	3.2683E+01	5.7926E+00	4.2760E+05	3.9937E+05	9.0905E+01
	30	Avg	6.0076E+06	9.3139E+06	1.4725E+06	1.6164E+09	2.2004E+06	2.1866E+03	**2.1780E+02**	2.2013E+03	2.4617E+03	2.5499E+03
		SD	7.6144E+06	6.5517E+06	1.0637E+06	8.1713E+08	1.1240E+06	5.4869E+01	2.5005E+00	2.3885E+02	2.9539E+02	2.6014E+02
	50	Avg	6.9927E+07	9.0731E+07	2.7144E+07	8.7175E+09	2.4682E+07	6.0624E+05	**8.2966E+02**	7.0584E+05	7.2011E+05	6.1816E+05
		SD	1.9852E+07	3.2599E+07	5.8046E+06	2.1223E+09	5.9167E+06	1.0765E+04	1.5298E+03	1.3725E+05	8.9903E+04	1.7907E+04
**Rank**	**10**	**(l/t/w)**	10/0/0	10/0/0	10/0/0	10/0/0	10/0/0	8/1/1	8/0/2	10/0/0	10/0/0	3/1/6
	**30**	**(l/t/w)**	10/0/0	10/0/0	10/0/0	10/0/0	10/0/0	9/1/0	8/0/2	10/0/0	10/0/0	2/1/7
	**50**	**(l/t/w)**	10/0/0	10/0/0	10/0/0	10/0/0	10/0/0	9/0/1	7/0/3	10/0/0	10/0/0	4/0/6

#### 5.3.1 Exploration and exploitation evaluation

The exploitative ability of algorithms is evaluated using unimodal functions, while the exploratory capability of algorithms is evaluated using multimodal functions. The exploitative and explorative qualifications of MMKE were evaluated and compared with comparative algorithms using these two kinds of test functions, which are detailed in the following:

As shown in Table 3, the MMKE algorithm has a significantly improved performance over MKE in gaining more accurate results for unimodal functions in dimensions 10, 30, and 50. This is mostly because the MKE-TVP and BTVP use the evolution strategy that is mostly exploitative since the best monkey or best-history archive of monkeys is selected to guide the search. In dimension 50, this is because of the usage of BTVP and RTVP, which assist in escaping from local optima while still preserving diversity. As a result, the MMKE algorithm exploits the optimum solution more efficiently than the MKE algorithm and other comparative algorithms.

As per the results stated in Table 4, MMKE is capable of producing competitive results for simple multimodal functions, particularly those with dimensions 10 and 30. This experiment is carried out on Func 4-Func 10, where the number of local optima rises exponentially while increasing the function dimension. These results prove that the proposed MMKE algorithm is competitive in exploration. The RTVP’s preservation of diversity and extensive exploration of the search space is the primary reason for the adequate exploration of MMKE.

#### 5.3.2 Evaluation of local optima avoidance

Hybrid and composition functions are usually composed of a variety of unimodal and multimodal functions, making them more complex and challenging in the optimization process. Consequently, these functions are appropriate for evaluating the MMKE’s capability to maintain the balance of exploration and exploitation, resulting in the avoidance of local optima.

For hybrid functions, the results in Table 5 show that MMKE surpasses all other algorithms in all three dimensions and produces better results. Furthermore, Table 6 shows the results of MMKE compared to comparative algorithms used in solving composition functions. Results demonstrated that the MMKE algorithm achieves a good balance between exploration and exploitation, which increases the ability to avoid local optima in a given situation. In addition, since each TVP’s improved rate is considered when determining the portion size of sub-populations, a suitable balance between exploration and exploitation can be achieved.

#### 5.3.3 Convergence evaluation

**Fig 12:**
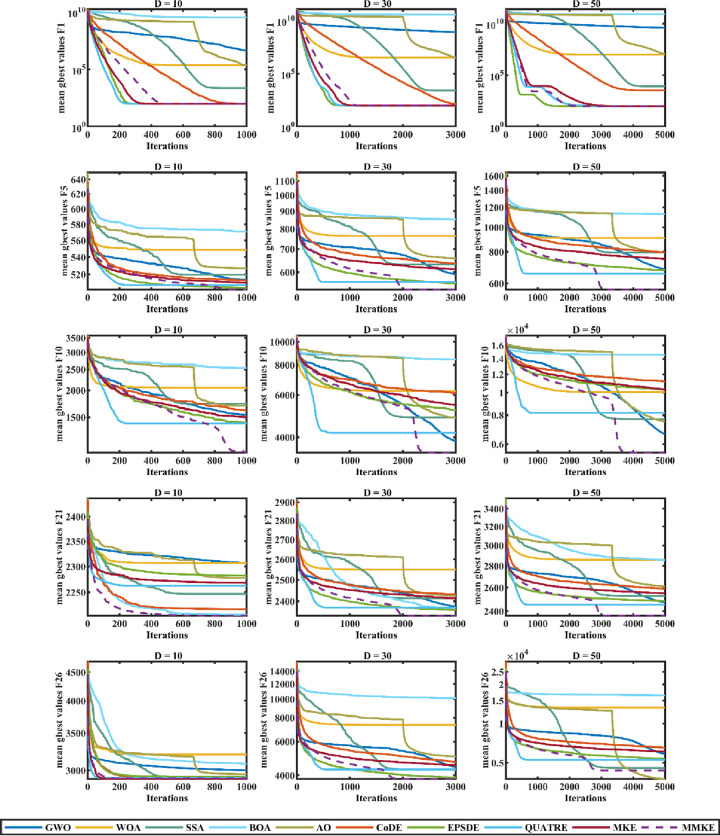
CEC 2018 convergence curves for selected functions with various dimensions.

This experiment aims to assess and compare the convergence behavior and speed of MMKE with the comparative algorithms. Fig 12 shows the convergence curves for the unimodal function Func 1, multimodal functions Func 5 and Func 10, and composition functions Func 21 and Func 26 on dimensions 10, 30, and 50. Each of these curves represents the mean of the best values in every generation over twenty runs for each algorithm.

As indicated by the curves in Fig 12, the MMKE demonstrates three convergence behaviors during the optimization process for test functions with diverse properties. First, there is a decreasing convergence in the early generations, in which an approximate optimal solution is found and maintained. The second behavior seen during the first half of the generations is faster convergence, and the estimate of the global optimum becomes increasingly accurate as the number of generations increases. Finally, the last behavior involves steady improvement of the solution until the last generations are reached. Based on the curves, it can be stated that the suggested MMKE algorithm is better capable of striking a balance between exploration and exploitation throughout generations than the comparative algorithms. The curves in Fig 12 indicate the competitive behavior of the MMKE algorithm while solving unimodal functions considering the performance assessment results presented in Tables 3–6 and Fig 12.

The MMKE algorithm is also superior to other comparative algorithms in which it achieves a faster convergence on multimodal and composition functions than comparative algorithms. Since the proposed algorithm uses a combination of best-obtained solutions in the BTVP and differences between random solutions, it achieves sufficient convergence, exploitation, and proper balance between exploration and exploitation. Additionally, the proposed algorithm maintains diversity throughout the optimization process by utilizing the differences between random solutions. The convergence curves presented in Fig 12 demonstrate that MMKE outperforms both the hybrid and composition functions, respectively. The MMKE’s gained results prove that the exploration and exploitation processes are appropriately balanced in both hybrid and composition functions. Furthermore, the proposed MMKE algorithm maintains the diversity essential for dealing with issues in complicated functions.

### 5.4 Discussion and limitations

This subsection discusses the primary advancements and reasons that make the proposed MMKE algorithm suited for tackling complex benchmark functions and global optimization problems with superiority over comparative algorithms. The qualitative results which are represented in Figs 9 and 10, by utilizing gbest-history, the BTVP can prevent premature convergence and entrapment in local optima and perform greater exploitation, as shown by the results of this investigation. Additionally, the RTVP is able to locate optimal solutions for hybrid and composition functions that can strike a balance between exploration and exploitation and prevent premature convergence. This TVP is vital for exploring and directing the optimal global search. The exploration and exploitation analysis that is presented in Fig 11 demonstrates that MMKE allocates a high proportion of its resources to the exploitation of unimodal test functions and a high percentage of its resources to the investigation of multimodal test functions. Using the winner-based distributing and reward rule distribution policy, the proposed MMKE algorithm successfully switch between exploration and exploitation in hybrid and composition test functions.

According to the results and curves shown in Table 3 and Fig 12, the MMKE algorithm is very competitive for unimodal and multimodal test functions by accurately converging to the promising area in terms of its ability to exploit and explore. It is attributable to the BTVP and RTVP, which promote the flow of information through the use of informative monkeys from the best-history archive as well as random monkeys. The experimental evaluations presented in Tables 4 and 5 and the convergence curves in Fig 12 demonstrate that utilizing TVP’s improved rate for determining the portion size of sub-populations enhances the probability of assigning the appropriate TVP. The experimental results demonstrate that the proposed MMKE algorithm competes with state-of-the-art evolutionary and swarm intelligence algorithms and has superior performance for solving unimodal, multimodal, hybrid, and composition test functions.

As with any research, ours has its limitations. Three TVPs were utilized in this investigation to inform the design of the winner-based distribution strategy, which will need to be revised for further trial vectors. The suggested MTDE’s performance may also suffer when the problem’s dimensions are exceedingly high. This restriction can be overcome by conducting preliminary experiments to establish the optimal archive size and policy for high-dimensional issues.

## 6. Statistical analysis

While the experimental assessment results compare the proposed MMKE algorithm’s overall performance to that of comparative algorithms, the statistical significance of the algorithms is not revealed. Thus, the Friedman test [111] is used to prove MMKE’s statistical superiority. The non-parametric test Friedman test (*F*_*f*_) is used to rank all algorithms according to their performance. This test is utilized to determine the MMKE and comparative algorithms ranking according to their obtained fitness by Eq (16),

$$F_{f}=\frac{12\times n}{k\times (k+1)}\left[\sum_{j}R_{j}^{2}-\frac{k{\times (k+1)}^{2}}{4}\right]$$ (16)

where *k*, *n*, and *R*_*j*_ are the number of algorithms, case tests, and the mean rank of the *j*^*th*^ algorithm, respectively. It scores each algorithm/problem pair from 1 (best outcome) to k (worst result) and then averages the rankings achieved across all problems to get the algorithm’s final rating.

In the Friedman test, the null hypothesis H0 which indicates there is no significant difference between the compared algorithms with p-value > 5%. The alternative hypothesis H1 assumed there is a significant difference between the results of the used algorithms for the 20 runs. This test scores each algorithm/problem pair from 1 (best outcome) to k (worst result) and then averages the rankings achieved across all problems to get the algorithm’s final rating. Better algorithms are identified by small ranks. The results of the Friedman rank test at a 95% confidence level are given in Table 7. According to Table 7, the p-value reached through the non-parametric test indicated the significance of the results and demonstrated the MMKE algorithm’s superiority on dimensions 10, 30, and 50 in comparison to state-of-the-art algorithms.

**Table 7:** Results of the Friedman test.

Algorithm	Dim	Func1	Func3	Func4	Func5	Func6	Func7	Func8	Func9	Func10	Func11	Func12	Func13	Func14	Func15	Func16		Func17
GWO	10	7.45	8.30	8.50	4.85	6.05	5.25	4.65	6.75	5.10	6.30	6.95	7.45	7.45	7.45	6.35		6.90
	30	9.00	7.90	7.90	4.10	6.00	4.55	3.65	6.00	2.55	7.55	7.95	6.85	7.40	7.30	4.15		5.20
	50	9.00	7.10	8.75	3.45	6.00	5.30	3.75	6.20	2.25	8.90	8.60	8.05	7.40	7.75	3.65		4.70
WOA	10	7.95	8.50	7.70	8.85	8.80	8.85	8.45	9.00	8.50	8.05	8.05	7.75	7.65	7.95	7.85		8.25
	30	7.40	9.70	8.35	8.95	9.00	9.00	8.80	8.80	8.05	8.40	8.50	7.35	8.80	8.00	8.90		8.50
	50	7.20	7.40	7.95	8.70	9.00	9.00	8.70	8.85	6.70	8.00	8.30	6.95	8.15	7.30	8.85		8.95
SSA	10	6.00	4.00	6.60	6.65	7.30	6.60	7.10	6.10	6.65	7.80	7.00	7.60	6.65	6.55	7.25		7.50
	30	5.90	**1.70**	6.15	6.00	7.45	4.90	6.15	7.55	4.80	6.50	6.15	7.15	6.10	7.05	6.20		6.80
	50	5.55	**1.00**	5.80	6.45	7.35	5.15	6.30	6.95	4.00	5.95	6.20	6.70	6.10	6.60	4.95		7.15
BOA	10	10.00	10.00	10.00	10.00	9.95	10.00	10.00	10.00	10.00	10.00	10.00	10.00	9.75	9.95	9.90		10.00
	30	10.00	9.10	10.00	10.00	10.00	10.00	10.00	10.00	10.00	10.00	10.00	10.00	9.95	10.00	10.00		10.00
	50	10.00	9.55	10.00	10.00	10.00	10.00	10.00	10.00	10.00	10.00	10.00	10.00	10.00	10.00	10.00		10.00
AO	10	8.60	7.20	7.20	7.75	7.90	7.55	7.25	7.90	6.10	7.85	8.00	7.20	8.50	8.10	7.50		7.10
	30	7.60	6.30	7.00	7.35	7.55	8.00	6.75	7.65	4.75	7.50	7.40	8.65	7.75	7.65	7.50		8.00
	50	7.80	6.65	6.90	6.90	7.65	7.95	7.10	8.00	3.55	6.95	6.90	8.30	8.35	8.35	7.55		7.45
CoDE	10	5.00	6.00	3.15	5.65	5.00	6.00	5.85	5.25	5.80	4.75	3.90	4.70	3.35	4.30	3.65		3.20
	30	5.10	4.75	5.40	6.75	5.00	6.70	7.10	4.65	8.40	4.50	**1.35**	3.95	3.50	3.50	4.60		3.85
	50	5.45	2.10	3.65	7.20	4.80	6.45	7.00	2.90	8.75	3.45	3.25	2.25	2.15	2.30	5.65		4.55
EPSDE	10	**2.00**	**2.00**	**1.15**	2.20	2.42	2.35	2.20	**2.50**	3.80	3.50	2.15	2.95	4.50	3.30	4.50		4.55
	30	3.40	2.55	**1.00**	2.50	**1.07**	2.30	2.25	1.88	5.65	2.60	4.40	3.50	2.65	3.70	4.45		5.00
	50	**1.00**	7.75	**1.50**	3.30	**1.00**	2.65	2.95	2.60	7.05	2.45	4.50	3.70	4.40	3.75	4.20		3.85
Quatre	10	**2.00**	**2.00**	1.85	3.45	2.15	3.25	4.15	**2.50**	3.55	3.20	2.80	2.80	3.98	3.90	3.95		3.95
	30	2.25	2.90	3.17	2.90	2.90	3.30	3.40	3.00	3.15	3.05	3.60	2.45	4.40	3.65	5.60		3.45
	50	2.70	3.00	3.25	2.50	4.10	2.50	2.65	4.90	4.45	4.20	2.50	**1.90**	4.10	4.45	4.90		4.00
MKE	10	4.00	5.00	3.95	4.35	3.38	4.00	4.30	**2.50**	4.40	2.45	4.05	3.20	2.00	1.95	2.60		2.27
	30	3.23	6.50	2.83	5.45	2.08	5.25	5.90	3.70	6.50	3.85	3.60	3.90	3.45	3.10	2.10		3.15
	50	4.00	6.25	3.85	5.50	2.00	5.00	5.55	3.52	7.15	3.70	3.30	4.35	3.35	3.40	3.90		3.25
MMKE	10	**2.00**	**2.00**	4.90	**1.25**	**2.05**	**1.15**	**1.05**	**2.50**	**1.10**	**1.10**	**2.10**	**1.35**	**1.18**	**1.55**	**1.45**		**1.27**
	30	**1.13**	3.60	3.20	**1.00**	3.95	**1.00**	**1.00**	**1.77**	**1.15**	**1.05**	2.05	**1.20**	**1.00**	**1.05**	**1.50**		**1.05**
	50	2.30	4.20	3.35	**1.00**	3.10	**1.00**	**1.00**	**1.07**	**1.10**	**1.40**	**1.45**	2.80	**1.00**	**1.10**	**1.35**		**1.10**
															**Avg. Rank**		**Overall Rank**	
Algorithm	Dim	Func18	Func19	Func20	Func21	Func22	Func23	Func24	Func25	Func26	Func27	Func28	Func29	Func30				
GWO	10	8.00	6.80	6.55	6.35	6.65	4.65	5.15	6.55	6.75	6.35	7.75	4.80	7.30	6.53		7	
	30	7.25	7.10	6.35	3.65	6.75	4.40	3.75	8.30	4.90	6.90	8.50	5.75	7.75	6.19		7	
	50	7.45	7.45	4.80	3.10	2.70	4.05	4.30	9.00	5.45	7.00	9.00	5.55	8.45	6.18		7	
WOA	10	7.20	8.75	8.30	8.05	8.35	8.50	6.90	7.80	8.25	8.65	7.00	8.75	7.00	8.13		9	
	30	8.30	8.70	8.50	8.90	7.95	9.00	8.95	8.35	8.65	8.60	7.20	8.90	8.45	8.52		9	
	50	8.45	8.35	9.00	9.00	6.90	9.00	9.00	7.40	9.00	9.00	7.95	9.00	8.55	8.30		9	
SSA	10	7.00	6.75	7.85	4.85	5.40	6.55	6.65	4.75	5.45	5.15	5.15	5.30	6.50	6.37		6	
	30	6.35	7.10	7.25	5.70	5.85	5.15	4.80	6.05	4.85	6.20	4.80	6.80	6.95	6.01		6	
	50	6.05	7.40	5.80	4.95	4.20	5.05	5.05	5.00	3.75	6.10	5.35	7.05	6.75	5.68		6	
BOA	10	10.00	9.80	9.60	9.50	10.00	10.00	10.00	10.00	9.95	10.00	9.95	9.95	10.00	9.94		10	
	30	10.00	10.00	10.00	10.00	10.00	10.00	10.00	10.00	10.00	10.00	10.00	10.00	10.00	9.97		10	
	50	10.00	10.00	10.00	10.00	10.00	10.00	10.00	10.00	10.00	10.00	10.00	10.00	10.00	9.98		10	
AO	10	7.80	7.90	7.50	6.65	8.00	8.20	7.85	6.65	6.65	7.45	7.55	7.55	6.80	7.53		8	
	30	8.10	7.10	7.00	6.40	4.95	8.00	7.95	5.05	5.95	8.05	6.40	7.90	6.85	7.14		8	
	50	8.05	6.80	6.25	7.60	4.05	8.00	7.95	7.10	**2.20**	7.80	6.70	7.85	6.25	7.00		8	
CoDE	10	3.25	3.90	2.00	3.80	3.25	5.00	3.50	3.45	5.85	2.50	3.55	5.35	2.50	4.26		5	
	30	**1.90**	4.70	2.95	7.30	3.80	6.60	6.80	4.75	6.35	3.45	3.25	4.95	2.75	4.78		5	
	50	**1.00**	2.95	3.80	7.05	7.75	6.90	4.05	3.45	7.50	2.30	2.70	4.35	2.50	4.42		5	
EPSDE	10	4.60	3.35	4.50	5.03	3.00	5.15	4.05	4.33	3.13	2.80	6.15	3.90	**1.00**	3.35		2	
	30	4.40	3.65	3.40	2.60	7.10	2.95	3.00	**1.00**	2.60	2.85	7.70	2.70	**1.00**	3.24		2	
	50	3.95	3.10	4.50	3.70	7.45	2.75	3.90	**1.30**	4.20	**1.00**	4.60	4.40	**1.00**	3.53		2	
Quatre	10	3.95	3.95	3.70	4.13	4.65	3.20	4.67	4.63	3.30	5.35	3.40	3.70	4.15	3.53		3	
	30	3.30	3.35	4.80	3.50	2.75	2.75	3.00	4.85	4.10	3.25	2.38	3.75	2.55	3.36		3	
	50	4.05	4.75	5.30	2.80	4.05	2.75	3.40	3.70	4.15	4.55	3.48	3.35	3.55	3.65		3	
MKE	10	1.75	2.60	3.30	4.88	3.80	2.65	4.40	4.55	3.30	4.53	2.90	4.55	6.05	3.57		4	
	30	3.25	2.10	2.95	5.90	4.55	5.15	5.75	3.55	5.65	2.95	3.30	2.55	4.25	4.02		4	
	50	3.55	3.10	4.25	5.80	6.75	5.50	6.35	4.00	6.30	3.65	3.25	2.35	4.60	4.40		4	
MMKE	10	**1.45**	**1.20**	**1.70**	**1.77**	**1.90**	**1.10**	**1.82**	**2.30**	**2.38**	**2.23**	**1.60**	**1.15**	3.70	**1.80**		**1**	
	30	2.15	**1.20**	**1.80**	**1.05**	**1.30**	**1.00**	**1.00**	3.10	**1.95**	**2.75**	**1.48**	**1.70**	4.45	**1.78**		**1**	
	50	2.45	**1.10**	**1.30**	**1.00**	**1.15**	**1.00**	**1.00**	4.05	2.45	3.60	**1.98**	**1.10**	3.35	**1.86**		**1**	

## 7. Applicability of MMKE for solving engineering design problems

As discussed in previous sections, metaheuristic algorithms are extremely useful for solving real-world engineering [112–116]. This section includes five engineering problems that were used to study further the MMKE algorithm’s potential to address real-world engineering difficulties. MMKE and other comparative algorithms have been applied to the pressure vessel design [117], the welded beam design [118], the tension/compression spring [119], the three-bar truss [120], and the optimal power flow problems for the IEEE 30-bus system [121]. A detailed description of these problems can be found in S1 Appendix. The death penalty function is used to manage constraints in these problems such that solutions that violate any of the constraints are ignored. As a result, a large number increases the fitness value of solutions that disrupt one or more constraints [1]. Each algorithm is run 30 times individually in this experiment, with the maximum number of generations (*MaxGen*) and the population size (*N*) were considered *(Dim×10^4)/N* and 20, respectively. Also, in the last experiment, they set to 20, 50, and 200. For each problem, the design problem’s obtained values (DV), and the optimum value of the design problem’s objectives are tabulated in Tables 8–13.

**Table 8:** Comparison of variables and objective values for the problem of pressure vessel.

Algorithms	Variables’ optimum values				Optimal Cost
	*T*_*s*_	*T*_*h*_	*R*	*L*	
GWO	0.77871	0.38613	40.34139	199.71096	5890.888
WOA	0.79532	0.39676	40.75520	194.02393	5986.104
SSA	0.80423	0.39753	41.66972	182.02297	5931.400
AO	0.81699	0.40735	42.17072	175.9860	5992.254
CoDE	0.88184	0.44901	43.05134	168.19763	6532.407
EPSDE	0.77817	0.38465	40.31962	200.00000	**5885.333**
QUATRE	0.83117	0.41085	43.06589	165.00624	5982.244
MKE	0.77817	0.38465	40.31962	200.00000	**5885.333**
MMKE	0.77817	0.38465	40.31963	199.99990	**5885.333**

**Table 9:** Comparison of variables and objective values for the problem of welded beam.

Algorithms	Variables’ optimum values				Optimal Cost
	*h*	*l*	*t*	*b*	
GWO	0.20541	3.47884	9.03594	0.20577	1.72570
WOA	0.18995	3.99627	8.71047	0.22704	1.87153
SSA	0.20462	3.49445	9.03685	0.20573	1.72639
AO	0.19258	3.94571	9.27555	0.20620	1.81296
CoDE	0.20007	3.57545	9.22626	0.20670	1.77064
EPSDE	0.20573	3.47049	9.03662	0.20573	**1.72485**
QUATRE	0.20574	3.47030	9.03650	0.20574	1.72492
MKE	0.20573	3.47055	9.03655	0.20573	1.72488
MMKE	0.20573	3.47049	9.03662	0.20573	**1.72485**

**Table 10:** Comparison of variables and objective values for the problem of tension/compression spring design.

Algorithms	Variables’ optimum values			Optimal weight
	*d*	*D*	*N*	
GWO	0.051176	0.344493	12.058282	0.01268
WOA	0.054237	0.421179	8.314372	0.01278
SSA	0.050000	0.317217	14.055452	0.01273
AO	0.058054	0.510944	6.2556107	0.01421
CoDE	0.050264	0.319697	14.080980	0.01299
EPSDE	0.051689	0.356711	11.289385	**0.01267**
QUATRE	0.056957	0.497201	6.146576	0.01314
MKE	0.052466	0.375666	10.269711	0.01269
MMKE	0.051622	0.355105	11.384534	**0.01267**

**Table 11:** Comparison of variables and objective values for the problem of three-bar truss.

Algorithms	Variables’ optimum values		Optimal weight
	*x*_*1*_	*x*_*2*_	
GWO	0.78795	0.41032	263.8970
WOA	0.84126	0.27671	265.6145
SSA	0.78882	0.40784	263.8959
AO	0.77470	0.45499	264.6189
CoDE	0.78762	0.41128	263.9008
EPSDE	0.78868	0.40825	**263.8958**
QUATRE	0.78951	0.40588	263.8967
MKE	0.79191	0.39958	263.9435
MMKE	0.78868	0.40825	**263.8958**

**Table 12:** Comparison of variables and objective values for the OPF using IEEE 30-bus system for Case1.

	Case 1									
DVs	GWO	WOA	SSA	BOA	AO	CoDE	EPSDE	QUATRE	MKE	MMKE
P_G1_	174.565	178.805	165.059	176.503	166.312	178.469	176.622	177.006	176.861	177.004
P_G2_	48.246	43.572	44.385	41.873	52.911	48.748	48.169	48.688	48.750	48.725
P_G5_	23.502	23.058	24.002	16.762	21.586	21.065	21.372	21.306	21.230	21.303
P_G8_	19.791	20.004	24.540	21.811	26.316	19.331	21.545	21.128	21.155	21.073
P_G11_	13.300	14.994	15.025	19.036	13.957	12.317	12.275	11.892	12.014	11.852
P_G13_	12.970	12.000	18.323	16.421	12.000	12.298	12.067	12.000	12.007	12.005
V_G1_	1.084	1.079	1.092	1.091	1.100	1.099	1.099	1.100	1.100	1.100
V_G2_	1.066	1.059	1.075	1.071	1.057	1.083	1.085	1.088	1.088	1.087
V_G5_	1.032	1.035	1.046	1.054	0.992	1.061	1.064	1.062	1.061	1.059
V_G8_	1.035	1.035	1.049	1.054	0.999	1.065	1.063	1.069	1.069	1.068
V_G11_	1.052	1.063	1.083	1.085	1.078	1.099	1.095	1.100	1.100	1.100
V_G13_	1.040	1.038	1.059	1.049	1.039	1.095	1.094	1.100	1.100	1.100
T_11(6–9)_	0.989	1.019	1.029	0.920	1.036	0.996	1.012	1.044	1.040	1.030
T_12(6–10)_	1.086	0.994	0.977	1.043	0.996	0.974	0.971	0.900	0.907	0.914
T_15(4–12)_	1.000	1.021	1.066	1.066	1.100	0.976	1.007	0.986	0.990	0.989
T_36(28–27)_	0.997	1.015	1.012	1.037	0.971	0.996	0.978	0.966	0.969	0.965
Q_C10_	0.483	2.201	1.809	4.792	5.000	3.237	1.113	5.000	4.281	4.904
Q_C12_	1.222	3.965	1.774	0.082	0.306	3.254	4.034	5.000	4.660	4.818
Q_C15_	0.431	3.562	2.577	4.237	5.000	2.799	2.389	5.000	4.985	4.988
Q_C17_	2.215	4.597	2.819	1.072	0.000	4.920	2.799	5.000	4.860	4.990
Q_C20_	1.921	5.000	2.551	4.904	0.000	3.389	4.551	4.999	4.414	4.967
Q_C21_	3.730	1.875	1.371	1.239	0.000	4.836	3.643	5.000	4.904	4.994
Q_C23_	4.163	5.000	2.673	4.865	0.000	3.953	4.238	3.910	4.240	3.865
Q_C24_	2.905	5.000	3.279	3.912	5.000	3.722	4.603	5.000	4.901	4.999
Q_C29_	0.003	4.735	4.040	2.789	0.242	3.715	3.011	2.736	2.565	2.662
Cost ($/h)	801.617	801.817	803.305	805.140	806.287	799.436	799.373	799.071	799.087	**799.062**
Ploss(MW)	8.974	9.033	7.935	9.006	9.682	8.828	8.650	8.620	8.617	8.630
VD (p.u.)	0.387	0.468	0.600	0.761	0.456	1.526	1.437	1.860	1.808	1.832

P in MW, V in p.u., T in p.u., Q in MVAR.

**Table 13:** Comparison of variables and objective values for the OPF using IEEE 30-bus system for Case 2.

	Case 2									
DVs	GWO	WOA	SSA	BOA	AO	CoDE	EPSDE	QUATRE	MKE	MMKE
P_G1_	145.215	176.996	159.796	152.319	183.686	173.063	176.069	177.359	175.520	173.402
P_G2_	58.991	39.732	40.416	56.391	36.729	50.800	49.425	47.825	47.879	48.584
P_G5_	26.123	23.435	29.202	29.674	20.718	23.545	20.842	22.319	21.873	21.836
P_G8_	30.949	17.597	26.431	10.759	13.352	18.866	22.119	21.142	22.343	22.979
P_G11_	14.851	18.724	16.173	21.387	11.677	13.061	11.409	12.436	13.125	12.478
P_G13_	15.191	16.712	19.497	22.069	27.878	14.058	13.562	12.316	12.476	12.009
V_G1_	1.049	1.027	1.048	1.028	1.064	1.027	1.037	1.039	1.040	1.035
V_G2_	1.033	1.014	1.033	1.019	1.039	1.013	1.020	1.024	1.022	1.020
V_G5_	1.020	1.004	0.998	1.040	1.012	1.018	1.017	1.016	1.015	1.016
V_G8_	1.000	1.012	1.009	0.986	0.977	1.003	1.005	1.009	1.008	1.007
V_G11_	1.010	1.053	1.039	1.028	1.100	1.067	1.025	1.012	1.024	1.034
V_G13_	1.003	1.010	0.995	1.031	0.996	0.995	0.997	0.992	0.992	0.997
T_11(6–9)_	0.997	0.981	1.015	0.900	0.944	1.069	1.035	1.027	1.039	1.049
T_12(6–10)_	0.913	0.944	0.928	1.005	0.983	0.905	0.902	0.902	0.901	0.904
T_15(4–12)_	0.948	0.986	0.933	1.035	0.968	0.961	0.956	0.944	0.945	0.950
T_36(28–27)_	0.958	0.957	0.959	0.959	0.974	0.956	0.964	0.971	0.966	0.970
Q_C10_	1.967	0.357	3.498	2.670	0.742	3.418	3.102	4.634	1.609	4.685
Q_C12_	1.557	0.584	2.436	1.942	1.777	3.260	3.043	0.308	0.704	0.035
Q_C15_	4.142	3.343	2.599	1.837	0.495	3.559	3.987	4.854	4.352	4.531
Q_C17_	2.464	4.523	1.632	3.696	0.000	2.376	2.471	0.267	2.949	0.372
Q_C20_	2.620	3.869	2.186	2.631	0.038	3.735	4.724	4.968	4.902	4.993
Q_C21_	4.097	0.603	1.961	4.098	1.792	4.607	4.533	4.982	4.963	4.693
Q_C23_	1.089	2.762	2.245	4.324	4.221	4.973	4.794	4.996	4.872	4.865
Q_C24_	4.178	2.537	2.451	3.045	0.080	4.408	4.568	4.979	4.935	4.990
Q_C29_	4.387	2.130	2.307	0.642	5.000	2.772	2.390	2.715	2.132	2.535
Cost ($/h)	812.395	808.216	811.942	820.591	815.714	805.546	804.358	804.204	804.031	**803.182**
Ploss(MW)	7.920	9.796	8.116	9.198	10.641	9.994	10.027	9.997	9.817	9.887
VD (p.u.)	0.146	0.159	0.163	0.234	0.289	0.119	0.104	0.094	0.101	0.095

P in MW, V in p.u., T in p.u., Q in MVAR.

This experiment aims to investigate the stated hypothesis in real-life scenarios, whether using the MTV approach in the MKE algorithm enhances its performance in terms of local optima avoidance, premature convergence, and balanced equilibrant exploitation and exploration. Thus, four constrained engineering design problems and a medium-scale OPF with two cases are considered. Based on the gained results, not only the proposed MMKE algorithm does perform better in constrained small-scale problems, but also achieves excellent results on medium-scale OPF problem for both single and multi-objective cases. In engineering design problems, MMKE could obtain similar and even better results because the cooperation of the defined TVPs prevents premature convergence and local optima trapping. In addition, sufficient exploration and exploitation of RTVP and preservation of diversity lead to superior results in medium-scale OPF problem. As a consequence of these results, the proposed MMKE algorithm is competitive and can discover objective values that are equivalent to or better than those solutions found by comparative algorithms.

## 8. Conclusion and future works

There are many stochastic algorithms, including evolution-based metaheuristic algorithms, that are well-known and powerful in solving optimization problems. There are, however, certain deficiencies to these algorithms when they are applied to complex problems. In this paper, we employed the multi-trial vector approach to present an effective multi-trial vector-based monkey king evolution (MMKE) algorithm, which is an improvement over the evolutionary algorithm known as monkey king evolution (MKE). The evolution strategy used by the MKE resulted in premature convergence and an inadequate balance of exploration and exploitation. Thus, the MTV approach substituted the simple MKE evolution scheme, which employs a combination of various TVPs. By utilizing the MTV approach, the population is divided into several sub-populations using a winner-based distributing policy, where each subpopulation possesses its own TVP. In MMKE, two strategies, BTVP and RTVP, are corporate with the canonical MKE-TVP such that various problems can be tackled with distinct characteristics. Furthermore, as an additional advantage, MMKE uses the prior knowledge of the best individuals to avoid local optimums and premature convergence.

In order to evaluate the performance of our proposed algorithm, a variety of experiments were carried out using the CEC 2018 test suite. First, MMKE was qualitatively evaluated in visual analysis subsection, followed by two directions of trial vector impact analysis and exploration and exploitation analysis. The main purpose of this subsection is to illustrate the significant effect and convergence behavior of suggested trial vectors throughout the search process, as well as their explorative and exploitative potential. Next, in Subsection 5.3, the performance of MMKE was quantitatively assessed in terms of exploration and exploitation, local optimality avoidance, and convergence assessment in comparison to eight state-of-the-art algorithms. The results revealed the effectiveness of MMKE in achieving optimum global solutions with more stable convergence than other well-known published optimization algorithms.

Then, experimental results were statistically analyzed using the Friedman test. In addition to demonstrating MMKE’s statistical superiority over comparative algorithms, the statistical results also demonstrated that the MMKE algorithm guarantees the efficacy of explorations and maintains a balance between exploration and exploitation. Finally, we evaluated the applicability of the MMKE by solving four engineering design problems and the optimal power flow problem for the IEEE 30-bus system. MMKE has been able to provide superior solutions, both in terms of optimal objective function values and the number of function evaluations for these problems. MMKE shown significant performance advantages over other well-known optimization algorithms in engineering design problems and its ability to deal with various constraint problems.

For the purpose of providing a concise summary of the results gained via the performance assessment, Table 14 provides the overall effectiveness (OE) of the MMKE and comparative algorithms based on their total performance results presented in Tables 3–6. Therefore, it is imperative to calculate the algorithms’ overall effectiveness (OE) by Eq (17), where *N* and *L* are the number of functions and the total number of loser functions for each algorithm, respectively.


OverallEffectiveness(OE)=((N−L)/N)×100 (17)

**Table 14:** Comparison of MMKE and comparative algorithms in terms of the overall effectiveness (OE).

	GWO	WOA	SSA	BOA	AO	CoDE	EPSDE	QUATRE	MKE	MMKE
Dim = 10	0/29/0	0/29/0	0/29/0	0/29/0	0/29/0	1/27/1	2/25/4	0/25/4	0/28/1	21/4/4
Dim = 30	0/29/0	0/290	1/28/0	0/29/0	0/29/0	2/26/1	4/24/1	0/28/1	0/28/1	20/7/2
Dim = 50	0/29/0	0/29/0	1/28/0	0/29/0	0/29/0	3/26/0	6/23/0	0/29/0	0/29/0	19/10/0
Total (w/l/t)	0/87/0	0/87/0	2/85/0	0/87/0	0/87/0	6/79/2	12/72/5	0/82/5	0/85/2	60/21/6
OE	0%	0%	2.29%	0%	0%	9.19%	17.24%	5.74%	2.29%	**75.86%**

The following remarks can be concluded from the overall effectiveness of MMKE and comparative algorithms. Firstly, for all different dimensions 10, 30, and 50, the proposed MMKE algorithm is superior to other comparative algorithms. In addition, another important remark is that MMKE is not only efficient than swarm intelligence algorithms including GWO, WOA, SSA, BOA, and AO but also it is a serious competitor for the evolutionary algorithms consisting of CoDE, EPSDE, QUATRE, and MKE.

The following conclusions can be taken from the results of experimental performance assessment, statistical analysis, and solutions gained for engineering design problems:

- The proposed best-history trial vector producer (BTVP) and random trial vector producer (RTVP) enhance exploitation and exploration.
- In cooperation with the canonical MKE-TVP, the proposed BTVP and RTVP assist in improving the overall balance between exploration and exploitation. Thus, it becomes possible for the MMKE to escape the local optimality.
- The results derived from different qualitative and quantitative experiments conducted on diverse test functions with various characteristics along with statistical tests testify to the superior performance of the MMKE algorithm over the comparative algorithms.
- It has been shown that the MMKE algorithm effectively solves engineering problems.

The MMKE algorithm was proposed for the purpose of optimizing continuous single-objective optimization problems. For future research, several directions can be considered. MMKE can be adapted to handle binary and multi-objective problems depending on the problem to be solved to tackle discrete, multi-objective, and many-objective real-world optimization problems. Moreover, attempting to tackle problems in various fields, such as scheduling, image processing, feature selection, clustering, and community detection, is beneficial. Eventually, proposing the aggregate version of MMKE such that the TVPs benefit from the search strategies of other algorithms could be a valuable and advantageous contribution.

## Supporting information

S1 File Tables of CEC2018 results. (XLSX)

S2 File Tables of engineering results. (XLSX)

S3 File Tables of OPF results. (XLSX)

S1 Appendix (DOCX)
