# Supplementary material for: MMKE: Multi-trial vector-based monkey king evolution algorithm and its applications for engineering optimization problems
Source: PLoS One. 2023 Jan 3;18(1):e0280006. doi: 10.1371/journal.pone.0280006 (PMC9810208; doi:10.1371/journal.pone.0280006)
Supplement: S1 Appendix — (DOCX) [file pone.0280006.s004.docx]

Appendix

- **Pressure vessel problem**

In the pressure vessel issue, as shown in Fig A.1, the aim is to take the lowest possible cost for the vessel's shaping, material, and welding [[117](#_ENREF_117)]. The mathematical description of the issue, as well as its limitations, are provided in Eq (A.1).

| Consider | $\vec{x}=\left[ x_{1}x_{2}x_{3}x_{4} \right]=[T_{s}T_{h} R L]$ | (A.1) |
| --- | --- | --- |
| Minimize | $f\left( \vec{x} \right)=0.6224x_{1}x_{3}x_{4}+1.7781x_{2}x_{3}^{2}+3.1661x_{1}^{2}x_{4}+19.84x_{1}^{2}x_{3}$ |  |
| Subject to | $g_{1}\left( \vec{x} \right)=-x_{1}+0.0193x_{3}\leq0$, |  |
|  | $g_{2}\left( \vec{x} \right)=-x_{2}+0.00954x_{3}\leq0$, |  |
|  | $g_{3}\left( \vec{x} \right)=-{\pi x_{3}^{2}x}_{4}-\frac{4}{3}\pi x_{3}^{3}+1.296.000\leq0$, |  |
|  | $g_{4}\left( \vec{x} \right)=x_{4}-240\leq0$ |  |
| where | $0\leq x_{i}\leq100. i=1. 2$ |  |
|  | $10\leq x_{i}\leq200. i=3.4$ |  |


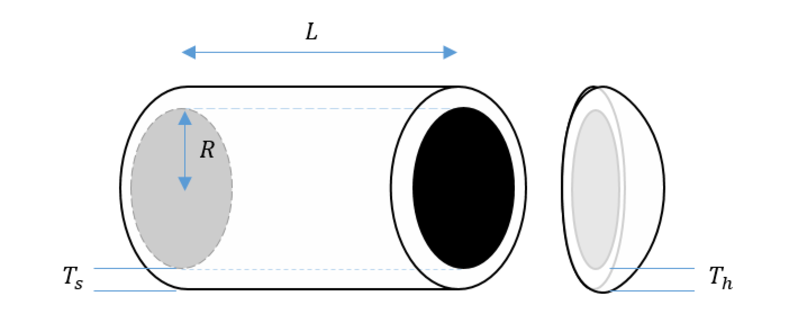


Fig A.1. Pressure vessel design problem.

- **Welded beam problem**

It is the aim of this design challenge [[118](#_ENREF_118)] to model a welded beam with the lowest possible production cost, as shown in Fig A.2. Eq (A.2) represents the mathematical description of this issue.

| Consider | $\vec{x}=\left[ x_{1}x_{2}x_{3}x_{4} \right]=[h l t b]$ | (A.2) |
| --- | --- | --- |
| Minimize | $f\left( \vec{x} \right)=1.10471x_{1}^{2}x_{2}+0.04811x_{3}x_{4}\times(14.0+x_{2})$ |  |
| Subject to | $g_{1}\left( \vec{x} \right)=\tau\left( \vec{x} \right)-\tau_{max}\leq0$, |  |
|  | $g_{2}\left( \vec{x} \right)=\sigma\left( \vec{x} \right)-\sigma_{max}\leq0$, |  |
|  | $g_{3}\left( \vec{x} \right)=\delta\left( \vec{x} \right)-\delta_{max}\leq0$, |  |
|  | $g_{4}\left( \vec{x} \right)=x_{1}-x_{4}\leq0$ |  |
|  | $g_{5}\left( \vec{x} \right)=P-P_{c}\left( \vec{x} \right)\leq0$ |  |
|  | $g_{6}\left( \vec{x} \right)=0.125-x_{1}\leq0$ |  |
|  | $g_{7}\left( \vec{x} \right)=1.10471x_{1}^{2}+0.04811x_{3}x_{4}\times\left( 14.0+x_{2} \right)-0.5\leq0$ |  |
| where | $0.1\leq x_{i}\leq2. i=1. 2$ |  |
|  | $0.1\leq x_{i}\leq10. i=3.4$ |  |


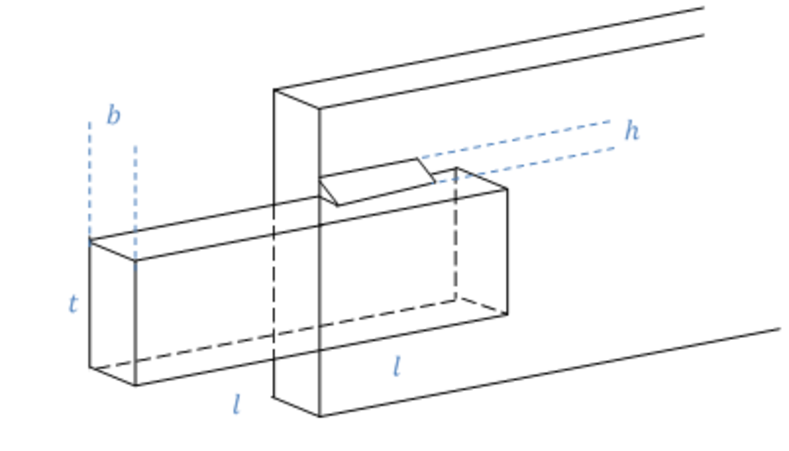


Fig A.2. Welded beam design problem.

- **Tension/compression spring design problem**

According to [[119](#_ENREF_119)], the purpose of this problem is to reduce the weight of the tension/compression springs, as depicted in Fig A.3. Eq (A.3) depicts the formulation for the problem.

| Consider | $\vec{x}=\left[ x_{1}x_{2}x_{3} \right]=[d D N]$ | (A.3) |
| --- | --- | --- |
| Minimize | $f\left( \vec{x} \right)=(x_{3}+2){x_{2}x}_{1}^{2}$ |  |
| Subject to | $g_{1}\left( \vec{x} \right)=1-\frac{x_{2}^{3}x_{3}}{71785x_{1}^{2}}\leq0$, |  |
|  | $g_{2}\left( \vec{x} \right)=\frac{{4x}_{2}^{2}-x_{1}x_{2}}{12566(x_{2}x_{1}^{3}-x_{1}^{4})}+\frac{1}{5108x_{1}^{2}}-1\leq0$, |  |
|  | $g_{3}\left( \vec{x} \right)=1-\frac{140.45x_{1}}{x_{2}^{2}x_{3}}\leq0$, |  |
|  | $g_{4}\left( \vec{x} \right)=\frac{x_{1+}x_{2}}{1.5}-1\leq0$ |  |
| where | $0.05\leq x_{1}\leq2.00$, |  |
|  | $0.25\leq x_{2}\leq1.30$, |  |
|  | $2.00\leq x_{3}\leq15.0$ |  |


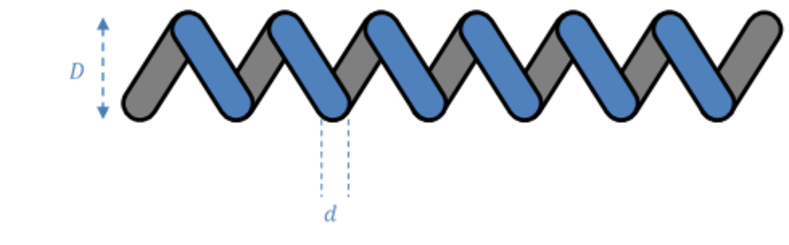


Fig A.3. Tension/compression spring design problem.

- **Three-bar truss problem**

This issue aims to reduce the weight of a three-bar truss while maintaining the restrictions [[120](#_ENREF_120)]. The problem's mathematical formulation and figure are presented in Eq (A.4) and Fig A.4.

| Consider | $\vec{x}=\left[ x_{1}x_{2} \right]=[A_{1} A_{2}]$ | (A.4) |
| --- | --- | --- |
| Minimize | $f\left( \vec{x} \right)=\left( 2\sqrt{2}x_{1}+x_{2} \right)*l$ |  |
| Subject to | $g_{1}\left( \vec{x} \right)=\frac{\sqrt{2}x_{1}+x_{2}}{\sqrt{2}x_{1}^{2}+2x_{1}x_{2}}P-\sigma\leq0$, |  |
|  | $g_{2}\left( \vec{x} \right)=\frac{x_{2}}{\sqrt{2}x_{1}^{2}+2x_{1}x_{2}}P-\sigma\leq0$, |  |
|  | $g_{3}\left( \vec{x} \right)=\frac{1}{\sqrt{2}x_{2}+x_{1}}P-\sigma\leq0$, |  |
| where | $0\leq x_{1}.x_{2}\leq1$, |  |
|  | $l=100cm. P=2kN/cm^{2}$, $\sigma=2kN/cm^{2}$ |  |


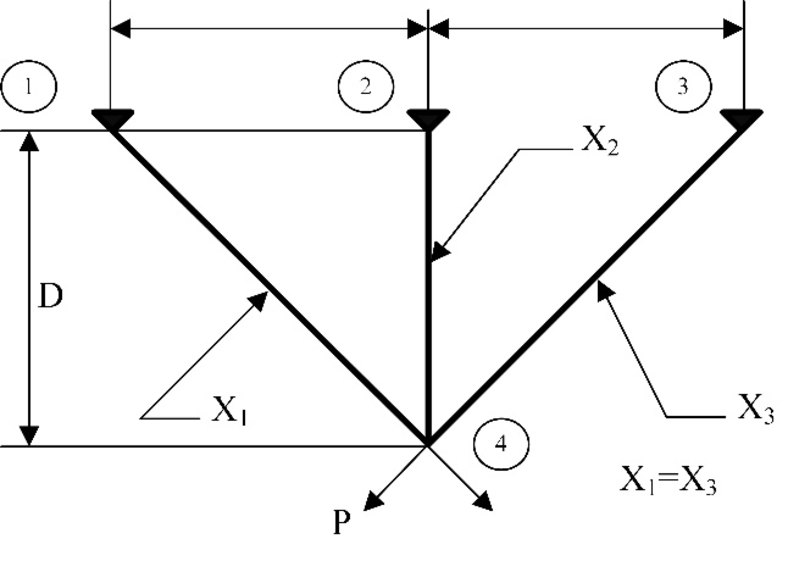


Fig A.4. Three-bar truss problem.

- **Optimal power flow problem for IEEE 30-bus system**

Six generators, four transformers, and nine shunt VAR compensation buses are included in the IEEE 30-bus system seen in Fig A.5. [[121](#_ENREF_121)]. The lower and upper bound values of decision variables are set as follows: shunt VAR compensations 0.0 and 0.05 p.u, transformer tap 0.9 and 1.1 p.u, and voltages for all generator buses 0.95 and 1.1 p.u.

**Case 1: Minimizing the cost of fuel**

The goal of Case 1 (*f_1_*) is to reduce the fuel costs of the generators as much as possible using Eq (A.5).

|  | (A.5) |
| --- | --- |

Where on the *i^th^* generator, the cost coefficients are considered *c_i_* in S/MW^2^h, *b_i_* in $/MWh_,_ and *a_i_* in $/hr.

**Case 2: Improving the voltage profile**

The purpose of Case 2 (*f2*) is to reduce the fuel cost and voltage variation as much as possible using the equation Eq (A.6) by considering *W_v_* = 200.

| $f_{2}=\sum_{i=1}^{NG} (a_{i}+b_{i} P_{Gi}+c_{i} P_{Gi}^{2})+W_{v}\sum_{i=1}^{NL} \left\vert V_{i}-1.0\right\vert$ | (A.6) |
| --- | --- |


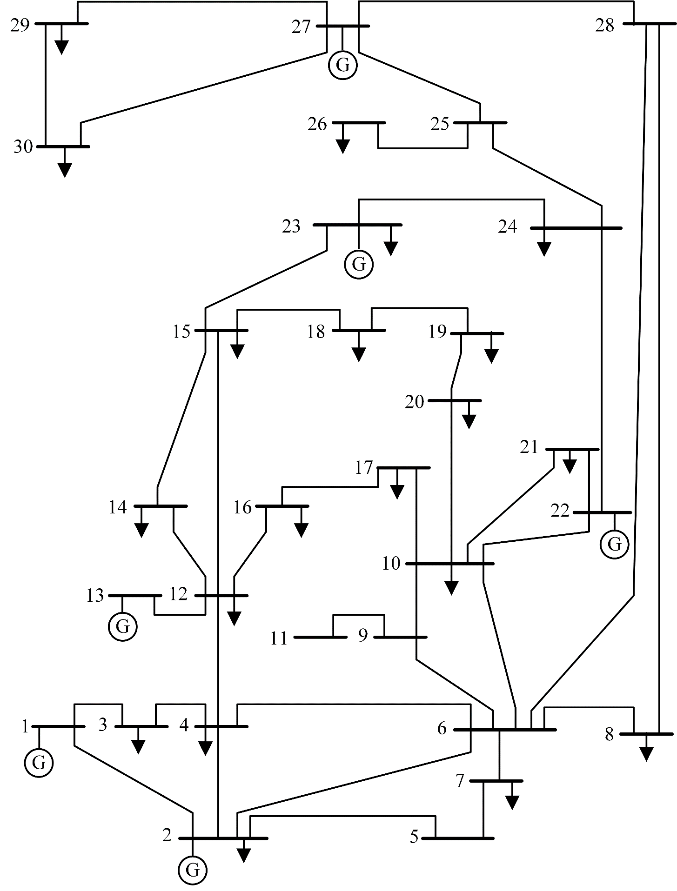


Fig A.5. IEEE 30-bus test system single-line diagram
